# Cross-protective human antibodies against the Mpox virus discovered through structure-guided screening

**DOI:** 10.1038/s41421-026-00916-2

**Published:** 2026-09-01

**Authors:** Dongdong Sun, Zihan Jia, Hongyu Han, Nan Zhang, Yiying Guo, Ruitian Hou, Wenli Zhao, Haiyan Yu, Lulu Wang, Yuhui Wang, Jun Dai, Hang Shang, Yamin Liu, Ding Li, Wenhui Tao, Zixian Sun, Ying Li, Zhiyu Ni, Wei Zheng, Haisheng Yu, Yu Guo

**Affiliations:** 1 State Key Laboratory of Medicinal Chemical Biology and College of Life Sciences, AAIS, Nankai University, Tianjin, China; 2 Guangzhou Laboratory, Guangzhou, Guangdong China; 3 Guangzhou Key Laboratory of Clinical Pathogen Research for Infectious Diseases, Institute of Infectious Diseases, Guangzhou Eighth People’s Hospital, Guangzhou Medical University, Guangzhou, Guangdong China; 4 College of Pharmacy and Drug Discovery Center for Infectious Disease, Nankai University, Tianjin, China; 5 College of Veterinary Medicine, Shanxi Agricultural University, Taigu, Shanxi China; 6 NITFID, School of Statistics and Data Science, AAIS, LPMC, and KLMDASR, Nankai University, Tianjin, China; 7 Tianjin Second People’s Hospital, Tianjin, China; 8 Central Laboratory, Hebei Collaborative Innovation Center of Tumor Microecological Metabolism Regulation, Affiliated Hospital of Hebei University, Baoding, Hebei China; 9 Frontiers Science Center for New Organic Matter, Nankai University, Tianjin, China

**Keywords:** Autoimmunity, X-ray crystallography

## Abstract

Mpox virus (MPXV) poses an increasing global health threat, as underscored by two World Health Organization declarations of Public Health Emergencies of International Concern, particularly after the emergence of a novel Clade Ib strain that exhibited high human-to-human transmissibility in the Democratic Republic of the Congo. However, the treatment options for MPXV infection remain extremely limited. To address this unmet need, we established an integrated platform combining single-cell transcriptomics and deep learning-based structural prediction to discover effective human monoclonal antibodies against MPXV. By integrating computational prediction with experimental validation, we identified five neutralizing antibodies targeting the following distinct viral forms: BA345, MA42, and MA49, which engage the extracellular enveloped virus-associated A35R glycoprotein; BAL31, which binds intracellular mature virus (IMV) protein A29L; and HB05, which targets IMV antigen H3L. Importantly, the elite monoclonal antibody BA345 conferred effective protection against MPXV and vaccinia virus both in vitro and in vivo. Combined in silico structure prediction and X-ray crystallography revealed a highly conserved epitope shared across orthopoxviruses. Surface plasmon resonance measurements revealed nanomolar equilibrium dissociation constants of BA345 for A35R homologs, corroborating its cross-reactive, broad-spectrum neutralizing activity against orthopoxviruses. Moreover, the BA345/BAL31 and MA49/BAL31 antibody cocktails developed in this study conferred robust therapeutic protection in MPXV-infected animals, substantially reducing disease severity and viral load. Our findings not only establish a practical paradigm for antibody discovery through the integration of deep learning-driven structure prediction with single-cell multiomics but also inform next-generation biodefense countermeasures against MPXV and related orthopoxviruses.

## Introduction

Infection with the mpox virus (MPXV), a member of the Orthopoxvirus genus^1^, has escalated into a global health crisis, with outbreaks extending far beyond its historical endemic zones in West and Central Africa^2,3^. Initially confirmed as a human-affecting pathogen in 1970 in the Democratic Republic of the Congo^4,5^, MPXV was responsible only for localized outbreaks until 2022, when a surge of cases in Europe signaled the start of a worldwide epidemic^6,7^. By August 2023, 113 countries had reported 88,600 confirmed cases of MPXV infection and 152 deaths (for a case fatality rate of 0.17%)^6,8^. The emergence of a novel Clade Ib variant in September 2023, with 18,737 suspected cases and 617 deaths (case fatality rate of 2.57%) reported across 13 African nations by August 2024^9,10^, further intensified the crisis, prompting the World Health Organization to redeclare a Public Health Emergency of International Concern (PHEIC) on August 14, 2024^11^.

Despite the increasing global incidence of mpox (formerly known as monkeypox), no vaccines or antiviral therapies have been specifically approved for this disease. Existing vaccines^12^, such as ACAM2000 and IMVAMUNE^3,13^, which were developed for preventing smallpox, are associated with adverse reactions and have been approved only for specific populations^14^, particularly those in endemic regions^15,16^. While the MPXV mRNA vaccine candidate BNT166 induces protective immunity in multiple animal models of infection^17^, it has not yet progressed beyond phase I/II clinical trials in humans^18^. Additionally, monoclonal antibody (mAb) therapies, which achieved great success during the COVID-19 pandemic^19–21^, offer a promising avenue; however, no mAb-based treatments have received emergency use authorization for mpox. The discovery and structural characterization of MPXV-specific mAbs remain an active area of research^22^.

MPXV exists in two infectious forms, the intracellular mature virus (IMV) and the extracellular enveloped virus (EEV)^23,24^, each presenting distinct neutralization targets. The primary IMV surface proteins include A29L^25^, H3L^26^, M1R^27,28^, and E8L^29–31^, whereas the key EEV proteins include A35R^19,32^ and B6R^33^. Recent studies of antibodies targeting B6R and M1R have demonstrated significant efficacy^33,34^. Notably, they may not fully prevent EEV release and spread, a critical aspect of the pathogenicity of MPXV driven by A35R^35–37^. The unique role of A35R in EEV formation and its interaction with B6R suggest that targeting A35R could disrupt a broader range of viral functions, potentially blocking cell-to-cell dissemination more effectively than existing therapies do^38–41^. This could address an unmet need to prevent viral immune evasion, a limitation of mAbs focused solely on IMV or partial EEV neutralization.

During the preparation of this manuscript, two related studies on MPXV A35-targeting antibodies were reported in *Cell*^42,43^. Ju et al. demonstrated that two human anti-A35 monoclonal antibodies (mAbs 975 and 981) confer protection against MPXV in mouse and nonhuman primate models and resolved high-resolution cryogenic electron microscopy structures of the A35–fragment antigen-binding (Fab) region complexes^40^. Separately, Ward, B. M. et al. identified three high-affinity human mAbs (EV35-2, -6, and -7) that block viral spread in vitro and protect mice from lethal MPXV and vaccinia virus challenge via Fc-dependent and -independent mechanisms^39^. In this context, our work independently identifies and characterizes a distinct panel of anti-A35R antibodies. We highlight BA345 as an elite candidate targeting a highly conserved epitope and uniquely demonstrate its enhanced therapeutic potential when it is used in combination with anti-IMV antibodies. Together, our findings and these parallel reports not only validate A35R as a promising target but also highlight the power of targeted antibody development for effective countermeasures against mpox.

The discovery of fully human neutralizing antibodies has been advanced by single-cell technologies, including microfluidic droplet screening, single-cell RNA sequencing (scRNA-seq), and optofluidic systems^31,44,45^. These tools allow high-throughput B-cell receptor sequencing, rapid candidate identification, and accelerated clinical translation^46,47^, as demonstrated in response to Zika^48^, influenza^49^, and SARS-CoV-2^44^. Despite this progress, a major bottleneck remains: the labor-intensive expression and functional characterization of hundreds to thousands of candidate antibodies. This screening is particularly critical in infectious diseases, where optimal antibodies must demonstrate both high potency and broad-spectrum activity against diverse strains and variants.

Structural insights into antibody‒antigen complexes are critical for selecting antibody candidates, as epitope specificity and binding dynamics directly govern neutralization efficacy and breadth. However, accurately predicting such complexes remains a challenge for existing deep learning methods. Combining single-cell sequencing with AI-based structure prediction now enables the efficient identification of antibodies that balance potency, breadth, and resilience to evolving pathogens, all of which are key traits for next-generation therapeutics. Tools such as AlphaFold^46^ and RosettaFold^47^ have revolutionized protein structure prediction, as evidenced by their top critical assessment of structure prediction (CASP) rankings. Their utility extends beyond single-chain folding to modeling protein‒ligand interactions and nucleic acid binding, underscoring their broad impact on predictive biology. Although modeling antibody–antigen binding is inherently difficult, the ability of these AI tools to predict protein–protein complexes offers a compelling path for structure-based discovery. This potential motivated our integrated deep learning approach to define the A35R–mAb interaction for the mpox virus.

In this work, we aimed to develop broad-spectrum antibody therapeutics against MPXV using an integrated computational and experimental approach. To achieve this goal, we utilized EnsembleFold^50^, a deep learning framework built on our CASP15-winning DMFold algorithm^11,51,52^, to predict the three-dimensional (3D) structures of antibody‒antigen complexes targeting four MPXV surface proteins: A35R, B6R, A29L, and H3L. Structure‑guided prioritization was followed by experimental characterization of the top‑ranked candidates. We successfully isolated and characterized five high-affinity neutralizing mAbs from MPXV-convalescent donors. Importantly, the EnsembleFold-predicted antibody‒antigen interaction modes strongly agreed with the experimentally determined crystal structures, validating the accuracy of our computational platform. Among these candidates, BA345 emerged as the lead therapeutic antibody because of its recognition of conserved epitopes across multiple orthopoxviruses, including vaccinia virus, cowpox virus, and variola virus, suggesting broad cross-protective potential. We subsequently engineered an antibody cocktail therapeutic (BA345/BAL31 and MA49/BAL31) that targets both the IMV and EEV forms of the mpox virus, likely by targeting multiple viral epitopes to counter viral escape. This study establishes a robust framework that identifies specific mAbs as candidate mpox therapeutics while highlighting their broader applicability for discovering antiviral mAbs against diverse threats using cutting-edge wet-lab and computational biotechnologies.

## Results

### scRNA-seq and single-cell B-cell receptor sequencing (scBCR-seq) for identifying memory B cells (MBCs)

**Fig. 1:**
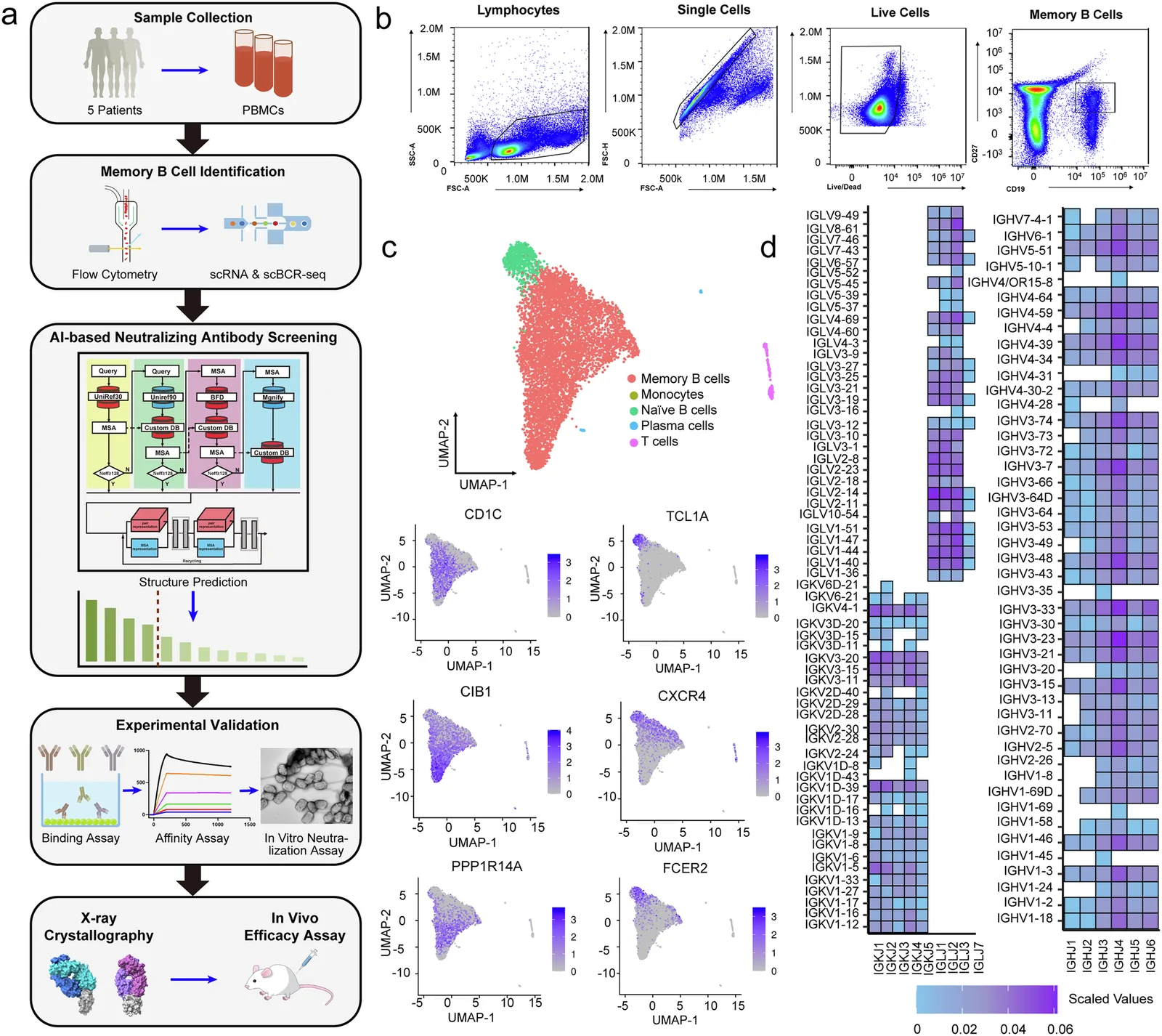
scRNA-seq and scBCR-seq for the precise identification of MBCs. **a** Overview of the experimental design. CD19^+^CD27^+^ MBCs were isolated from the peripheral blood of five individuals in the convalescent phase following mpox virus infection. scRNA-seq and scBCR-seq were performed to characterize the transcriptomic profiles and BCR repertoires of the individuals. Clonal lineage analysis was conducted to identify distinct B-cell clones, which were subjected to in silico screening through deep learning-based antibody‒antigen complex structure prediction. Finally, the candidate antibodies identified in this virtual screening step were validated in vitro and in vivo through functional assays to determine their neutralization ability. **b** Flow cytometry gating strategy for identifying MBCs from the PBMCs of Patient 5. Step 1: gating for the primary population, removing cell debris; Step 2: exclusion of clumped or sticky cells to ensure that only single cells remain; Step 3: removal of dead cells; Step 4: Gating for CD19^+^CD27^+^ B cells. **c** UMAP plots of 7132 B cells from Group 1. Orange and green correspond to memory and naïve B cells, which are characterized by the high expression of CD1C/CIB1/PPP1R14A and TCL1A/FCER2/CXCR4, respectively. **d** VDJ gene rearrangements for the heavy and light chains of Group 1.

The structural and functional characterization of many surface antigens on the two distinct morphological forms of MPXV remains limited, hindering the development of targeted mAbs. To directly obtain antibodies with known specificity for these viral antigens, we isolated CD19^+^CD27^+^ MBCs from the peripheral blood mononuclear cells (PBMCs) of five convalescent MPXV patients using flow cytometry. Such cells, derived from individuals who have successfully cleared MPXV infection, naturally produce antibodies against MPXV antigens. By isolating and characterizing these cells, we aimed to generate mAbs for structural and functional characterization against critical viral targets (Fig. 1a, b; Supplementary Fig. S1 and Table S1).

The sorted B cells were divided into three groups for scRNA-seq and scBCR-seq. The raw sequencing data were processed using the Cell Ranger bioinformatics platform to construct an immune repertoire, classify BCR clonotypes, and perform immune profiling. Uniform manifold approximation and projection (UMAP) analysis distinctly segregated MBCs from naïve B cells and non-B cells (Fig. 1c; Supplementary Figs. S2a, S3a). Critically, compared with naïve cells, MBCs exhibited significantly elevated expression of the markers CD1C, PPP1R14A, and CIB1. Conversely, the enrichment of TCL1A, FCER2, and CXCR4 in the naïve B cells was pronounced (Fig. 1c; Supplementary Figs. S2a, S3a). From 24,160 sequenced B cells across three libraries, 21,646 yielded productive V–J pairings. VDJ recombination analysis revealed dominant immunoglobulin gene usage: *IGHV3-23*, *IGHV4-59*, and *IGHV3-7* were the most frequent heavy chain genes, whereas *IGKV3-20*, *IGLV2-14*, and *IGKV4-1* were predominant in light chains (Fig. 1d; Supplementary Figs. S2b, S3b). Collectively, this transcriptional stratification confirms the successful enrichment of MBCs from convalescent donors, delineates the transcriptional profile of the MBC pool in MPXV convalescents, and reveals preferential immunoglobulin gene usage and molecular features of affinity maturation.

Clonotype screening of the isolated antibodies was conducted as described previously^19,53^. Clonotypes were initially filtered on the basis of the following criteria: frequency ≥ 1, expression of the IGHG1 isotype, presence of at least one MBC, and a somatic hypermutation (SHM) rate higher than 2%, which is indicative of affinity maturation. This stringent filtering, supported by prior studies^19^, aimed to enrich for clonotypes likely to represent bona fide antigen-experienced B cells. From the initial 21,646 productive V‒J pairings, this multistep process yielded 196 candidate antibody sequences for downstream characterization. These findings highlight the dominant immunoglobulin genes employed in the antibody response to MPXV, providing a basis for the subsequent deep learning-based discovery of neutralizing mAbs.

### Deep learning-based neutralizing antibody screening framework for MPXV

**Fig. 2:**
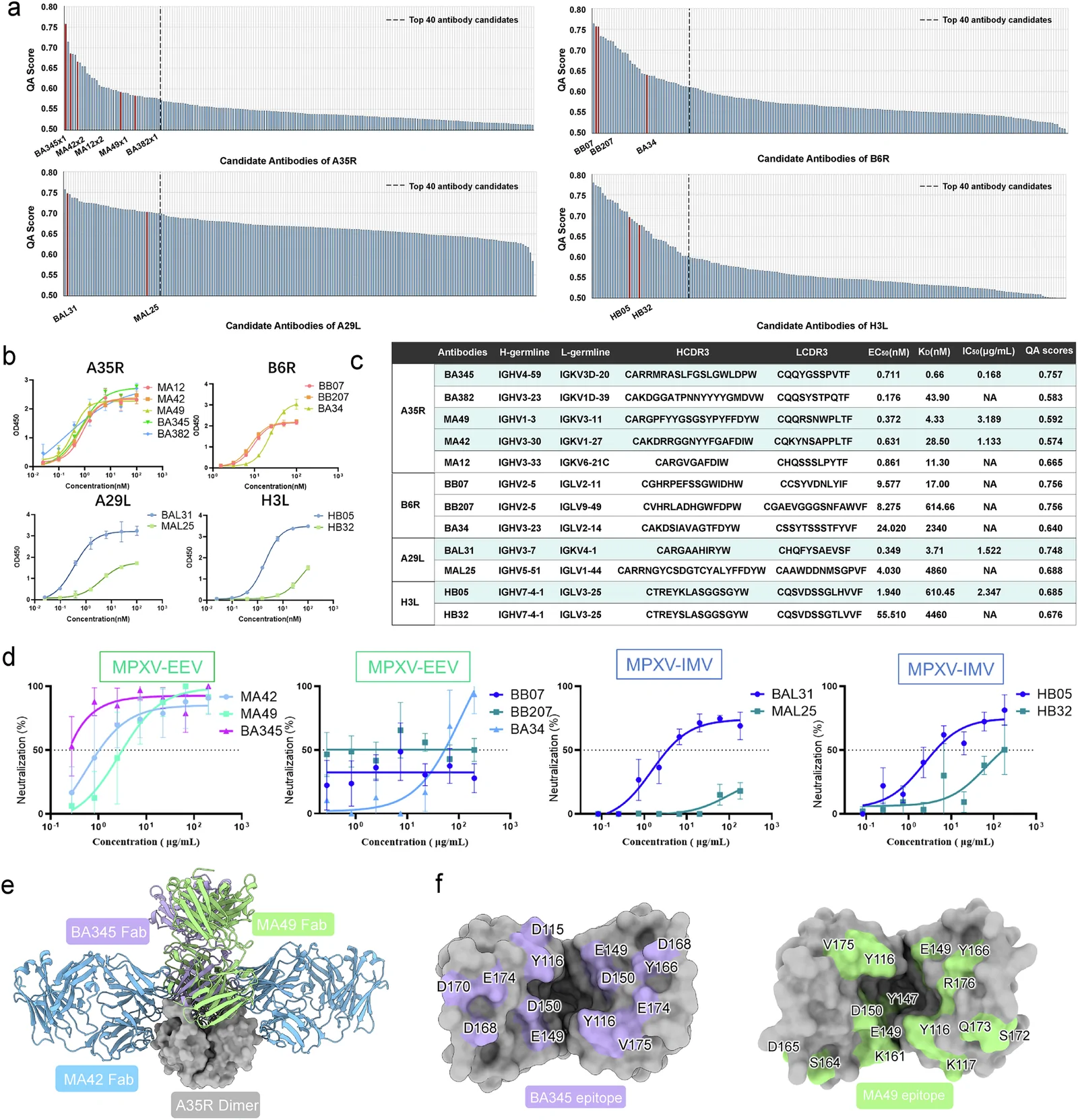
Discovery and characterization of neutralizing antibodies. **a** QA scores of 196 antibody candidates against the mpox virus proteins A35R, B6R, A29L, and H3L. Red bars represent antibodies that demonstrated high binding affinities in subsequent ELISA experiments. The dashed line separates the top 40 antibody candidates in terms of the QA score from the remaining antibodies. **b** ELISA activity of MA12, MA42, MA49, BA345, and BA382 against the mpox A35R protein; BB07, BB207, and BA34 against the mpox B6R protein; BAL31 and MAL25 against the mpox A29L protein; and HB05 and HB32 against the mpox H3L protein. **c** Sequence alignment of the HCDR3 and LCDR3 regions of the antibodies and a comparison of their EC_50_, *K*_D_, and IC_50_ values. NA indicates that no neutralizing activity or IC_50_ could be calculated. **d** Neutralizing potencies of the MA42, MA49, BA345, BB07, BB207, and BA34 antibodies against the EEV form of MPXV and of the BAL31, MAL25, HB05, and HB32 antibodies against the IMV form of MPXV were assessed with a plaque reduction neutralization test (PRNT). **e** Comparison of the structures of the MA42 Fab–A35R, MA49 Fab–A35R, and BA345 Fab–A35R complexes predicted by EnsembleFold. **f** Antibody footprint on A35R of the BA345–A35R and MA49–A35R complexes predicted by EnsembleFold.

To identify which of the above antibodies were capable of specifically targeting antigenic epitopes on the surface of the mpox virus, the EnsembleFold algorithm was employed to predict the 3D structures of the 196 candidate antibody complexes against four key viral surface antigens (A35R, B6R^27,34^, A29L^54,55^, and H3L^26^) on the basis of their critical roles in viral attachment/entry and to establish immunogenicity. Model confidence was evaluated using the quality assessment (QA) score, defined as a linear combination of the predicted template modeling score (pTM) and the interface-predicted TM score (ipTM), both computed by EnsembleFold. The pTM reflects the confidence in the overall global topology of the predicted structure, whereas the ipTM specifically assesses the predicted accuracy of interchain interfaces in multimeric models. For the A35R antigen, the highest-ranking complex achieved a QA score of 0.757; similar values were observed for the other antigens, with top QA scores of 0.764 for B6R, 0.757 for A29L, and 0.780 for H3L. Across all four antigens, the top 40 antibody candidates exhibited consistently high QA scores, ranging from 0.574 to 0.780, with moderate variability across candidates, indicating conservative selection to balance structural confidence and candidate diversity. With respect to the remaining candidates, the QA scores gradually decreased with decreasing rank and stabilized at approximately 0.604 for A29L and approximately 0.511 for the other three antigens, markedly lower than the scores observed for the top candidates. This distinct shift in the distribution of the QA score suggests that the top 40 antibody candidates are enriched with high-affinity binders and constitute a promising pool for the identification of effective neutralizing antibodies against the aforementioned viral antigens (Fig. 2a).

To experimentally validate the binding specificity of these computationally selected candidates, single‑well ELISA screening was simultaneously performed for all 196 candidate antibodies against the four antigens. Dose-response ELISA subsequently confirmed clones whose OD_450_ values were greater than 1.0. The results revealed that five antibodies bound to A35R, three to B6R, two to A29L, and two to H3L (Fig. 2b). Notably, all 12 antibodies that were experimentally validated as specific binders were indeed among the top 40 candidates ranked by EnsembleFold. This high degree of concordance between the computational predictions and the experimental screening corroborates the reliability of the AI-guided selection process. The specific binding affinities were determined using surface plasmon resonance (SPR), which confirmed high affinities for all five ELISA-positive antibodies (BA345, MA42, MA49, MA12, and BA382) to A35R, with equilibrium dissociation constant (*K*_D_) values of 0.66 nM (BA345), 11.30 nM (MA12), 4.33 nM (MA49), 28.50 nM (MA42), and 43.90 nM (BA382), respectively. BB07 and BB207 had significantly higher affinities for B6R than for BA34, whereas compared with MAL25, BAL31 exhibited superior binding to A29L. HB05 displayed a markedly greater affinity for H3L than HB32 did (Fig. 2c; Supplementary Fig. S4). Notably, BB207 and HB05 showed modest affinities, likely reflecting epitope-dependent limitations under the SPR conditions, yet their specific ELISA reactivity supported their use as bait antibodies in our QA‑guided selection. This observation suggests that our approach can identify functional binders across a range of affinities, although the lower value may reflect limitations in the recognition of certain epitopes under the conditions of our screening cascade. Together, these results confirm that our QA‑guided selection reliably identifies antibodies with both target specificity and sub‑nanomolar to low‑nanomolar binding affinities.

The neutralizing activities of these 12 antibodies against MPXV were evaluated in vitro. Given that A35R and B6R are specific to the EEV, whereas A29L and H3L are predominantly expressed on the IMV, the neutralizing efficacy was assessed accordingly using plaque reduction neutralization tests. In the presence of EEV, the antibodies BA345, MA42, and MA49 (anti-A35R) demonstrated robust neutralizing activities, with half-maximal inhibitory concentration (IC_50_) values of 0.168, 1.133, and 3.189 μg/mL, respectively. For IMV, BAL31 (anti-A29L) and HB05 (anti-H3L) similarly exhibited robust neutralizing activities, with IC_50_ values of 1.522 μg/mL and 2.347 μg/mL, respectively (Fig. 2c, d). These laboratory experiments confirmed that the structure-based ranking of EnsembleFold effectively highlighted functional neutralizers, as multiple high-affinity antibodies were identified within the top-ranked subset. By selecting only the top 40 highest-scoring antibodies from an initial pool of 196 for experimental characterization, the wet-lab workload was reduced significantly, streamlining the discovery pipeline and substantially lowering associated costs.

To define the structural elements underlying MPXV neutralization, the EnsembleFold framework was further employed to model the Fab regions of three lead antibodies (BA345, MA49, MA42) in complex with the A35R antigen (Fig. 2e; Supplementary Fig. S5). EnsembleFold generated high-accuracy predictions for all antibody‒antigen complexes, with structural analyses identifying BA345 as a priority therapeutic candidate owing to its precise targeting of conserved epitopes across orthopoxviruses (vaccinia virus, cowpox virus, and variola virus) (Fig. 2f; Supplementary Fig. S6). Structure-guided selection and phylogenetic conservation analyses suggested that BA345 is a robust, broad-spectrum poxvirus neutralizing antibody candidate. Biophysical characterization, including subnanomolar affinity and significant MPXV neutralization activity consistent with structural predictions, confirmed its high potential.

### Structural analysis of the MA42 Fab–A35R and MA49 Fab–A35R complexes

Building on the effective neutralization observed for MA42, MA49, and BA345, we determined the atomic-resolution crystal structures of their complexes with A35R to validate the computational predictions and define the molecular mechanisms underlying viral neutralization (Supplementary Tables S2, S3). Structural analyses revealed two different binding modes between MA42/MA49 and A35R. The MA42 Fab–A35R complex crystallizes with two complexes per asymmetric unit. Each MA42 Fab binds an A35R monomer, while the A35R glycoprotein forms a dimer along the crystallographic symmetry axis (Supplementary Fig. 3a). Structural analysis revealed a 481.7 Å^2^ interface between the A35R subunits, stabilized by three hydrogen bonds and an extensive hydrophobic network (Supplementary Fig. S7).

**Fig. 3:**
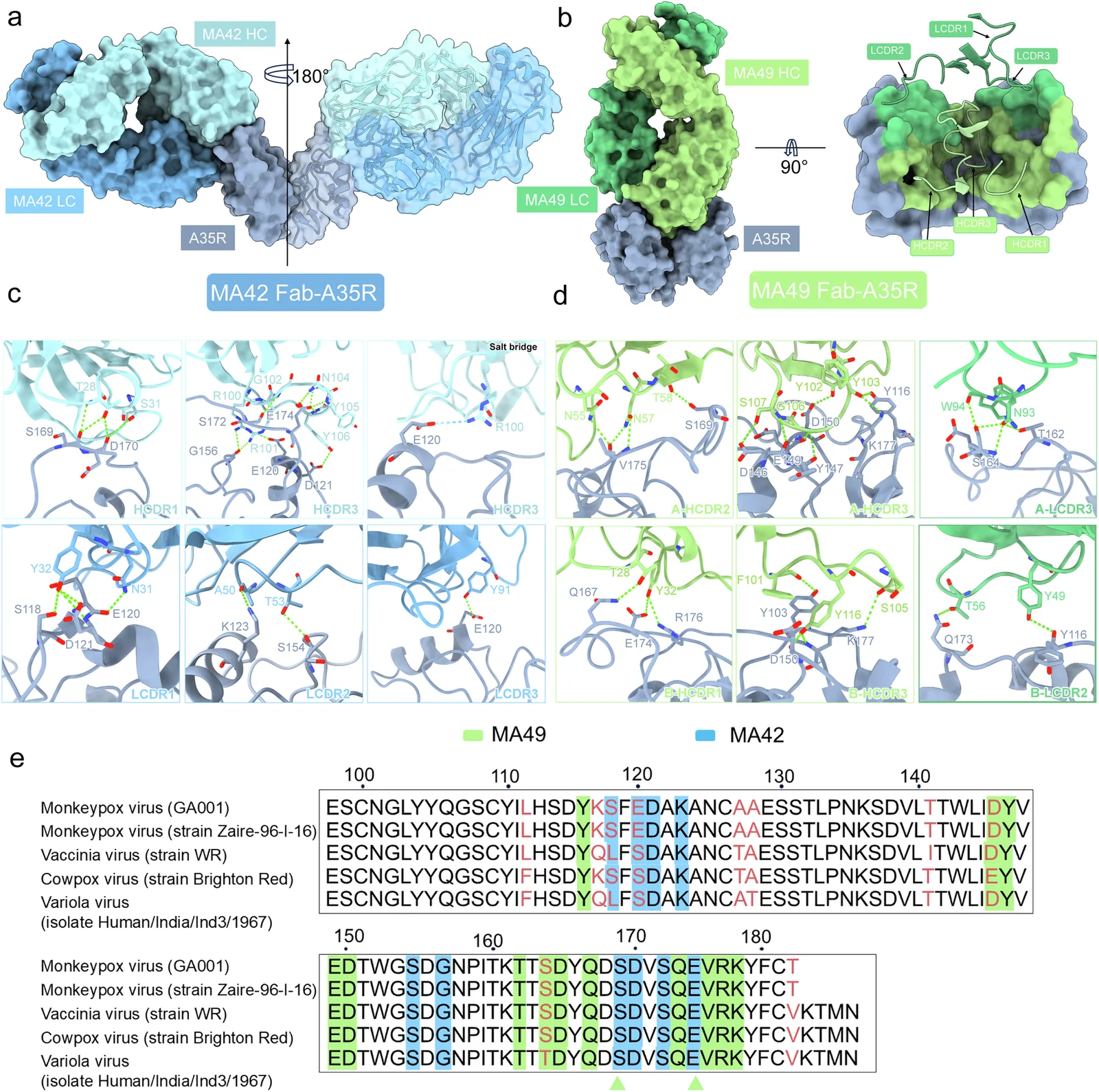
Structural analysis of the MA42 Fab–A35R and MA49 Fab–A35R complexes. **a** Overall structure of the MA42 Fab–A35R complex. The heavy chain (colored cyan) and light chain (colored darker blue) of the MA42 Fab region and A35R (colored gray) are displayed in surface representations. **b** Overall structure of the MA49 Fab–A35R complex. The heavy chain (colored olive drab) and light chain (colored green) of the MA49 Fab region and A35R (colored dark gray) are displayed in surface representations**. c** Interface representation of the binding of the MA42 Fab region to A35R. Hydrogen bonds are shown as green dashed lines, and salt bridges are shown as blue dashed lines. Residues are shown in stick representations with the same colors as in **a**. **d** Interface representation of the binding of the MA49 Fab region to A35R. Hydrogen bonds are shown as green dashed lines. The residues are shown in stick representation with the same colors as in **b**. **e** Sequence alignment of MPXV A35R from clade II and clade I and its orthologs in VACV, VARV, and CPXV. The amino acids marked in yellow contain mutations in A35R and its homologs across these viruses. The amino acids highlighted in blue and green significantly interact with the amino acids in MA42 and MA49, respectively. The green triangles indicate epitopes of A35R that are recognized by both MA49 and MA42.

MA42 engages the solvent-exposed α-helix (residues 119–128) and two flexible loop segments (residues 152–157, 167–176) of A35R through a multimodal binding interface with a total buried surface area (BSA) of 785.4 Å^2^ (Fig. 3c). Structural analyses indicated balanced heavy-light chain interactions: the heavy chain contributes a 494.3 Å^2^ BSA via HCDR1 and HCDR3, establishing a hydrogen bond network with A35R that is reinforced by a salt bridge between HCDR3 R100 and A35R E120, whereas the light chain contributes 291.1 Å^2^ of BSA through LCDR1, LCDR2, LFR3, and LCDR3 (Fig. 3c; Supplementary Table S5). Both chains exhibit extensive hydrophobic complementarity at the interface (Supplementary Table S6). To assess the broader applicability of MA42 beyond MPXV, we next examined the conservation of its A35R epitope across orthopoxvirus homologs. By aligning the A35R sequence with its vaccinia virus (VACV) and variola virus (VARV) counterparts (A33R proteins) and mapping the key contact residues identified in our crystal structure, we observed that several binding-critical positions (notably S118 and E120) are substituted in other orthopoxviruses (Fig. 3e) and are predicted to weaken hydrogen bonding and hydrophobic contacts with the MA42 paratope. These sequence variations were corroborated by comparative structural modeling, which revealed a reduced buried surface area and altered interface complementarity for MA42–A35R complexes. Thus, the homolog analysis revealed significant structural divergence at the MA42 binding site, explaining its limited cross‑reactivity and underscoring the antibody’s specificity for MPXV A35R.

Compared with MA42, MA49 has a different binding mode to the A35R dimer; specifically, the antibody engages the dimer through a single Fab region with significantly greater binding affinity, as corroborated by the SPR data (Fig. 3b; Supplementary Fig. S8a, b). Here, the heavy chain dominates the interaction interface, contributing a BSA of 1063.6 Å², whereas the light chain accounts for 370.6 Å² (Fig. 3d; Supplementary Table S5), for a total BSA of 1434.2 Å². Further analysis of the structural components underlying these BSAs revealed that MA49 forms an extensive network of hydrogen bonds with A35R, primarily involving the HCDR1, HCDR2, and HCDR3 regions of the heavy chain of MA49 and subunits A and B of A35R. Notably, the HCDR3 region of MA49 inserts into the central groove of the A35R dimer, establishing direct contact with residues from both subunits and thereby constituting the primary interaction interface (Supplementary Fig. S8e). In contrast, the light chain of MA49 forms fewer hydrogen bonds with A35R, primarily through the LCDR2 and LCDR3 regions. Collectively, both the heavy and light chains of MA49 establish comprehensive interactions with both subunits of A35R, contributing to the stability of the MA49–A35R complex (Fig. 3d; Supplementary Table S7).

To evaluate the potential cross-reactivity of MA49 across orthopoxviruses, we analyzed the evolutionary conservation of its epitope residues. Sequence alignments of A35R were performed across representative strains, including mpox (clades I/II), vaccinia (WR strain), cowpox, and variola (Ind3/1967). This analysis revealed that MA49 targets an epitope comprising residues Y116, Y147, E149, D150, T162, D165, S169, Q173, E174, V175, R176, and K177, which are evolutionarily conserved across all the examined orthopoxviruses. Conservative substitutions were observed at position D146E in cowpox and S164T in variola, preserving key physicochemical properties and suggesting that the binding capacity was largely retained despite potential attenuation (Fig. 3e; Supplementary Fig. S8d). This conservation pattern and the unique binding geometry of MA49 reflect its broad neutralization potential against orthopoxviruses.

### Structural analysis of the BA345–A35R complex

**Fig. 4:**
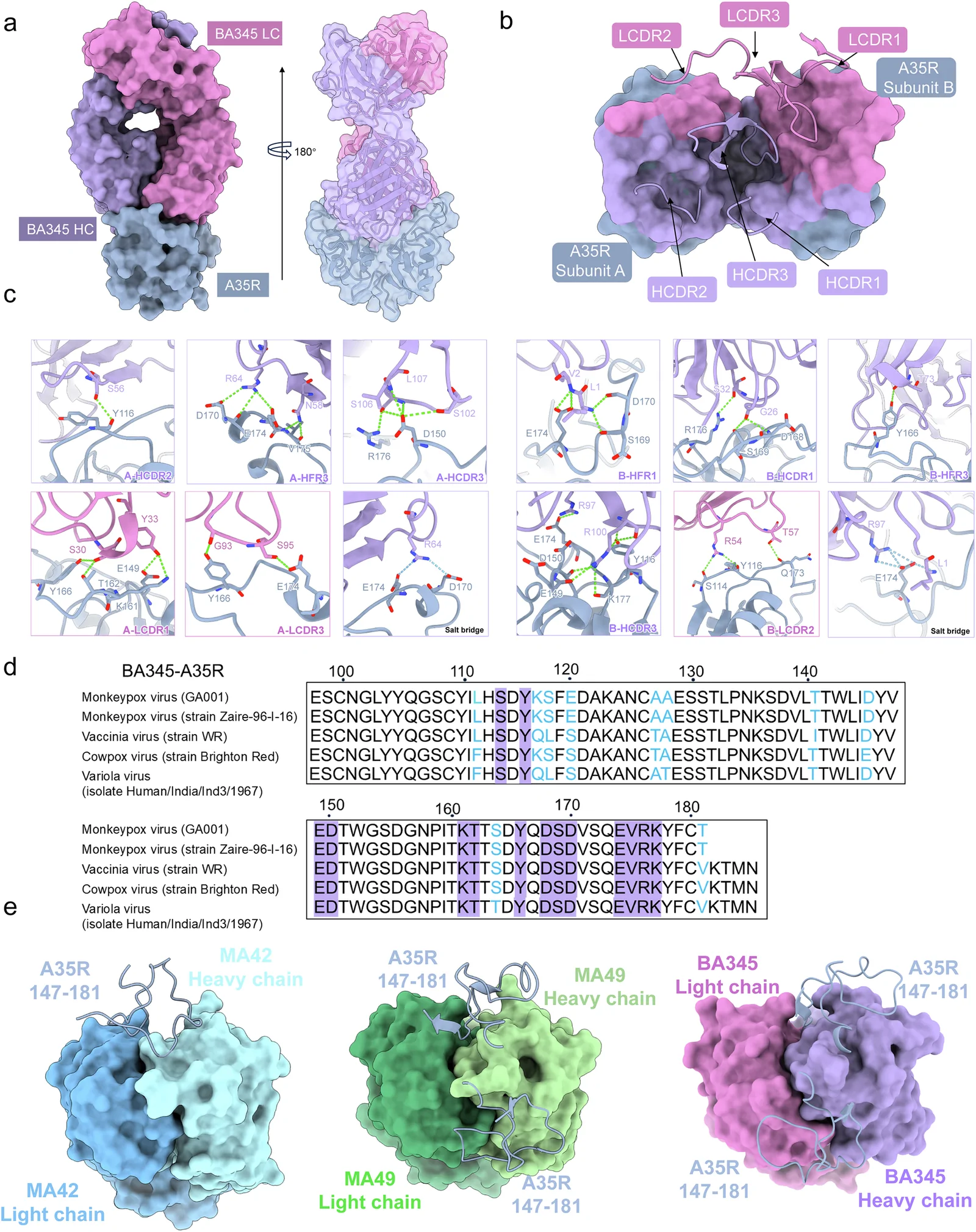
Structural analysis of the BA345 Fab–A35R complex. **a** Overall structure of the BA345 Fab–A35R complex. The heavy chain (colored purple) and light chain (colored pink) of the BA345 Fab region and A35R (colored dark gray) are displayed in surface representations. **b** Epitopes recognized by the BA345 Fab heavy chain and light chain are shown in surface representations. **c** Interface representations of the binding of the BA345 Fab region to A35R. Hydrogen bonds are shown as green dashed lines, and salt bridges are shown as blue dashed lines. The residues are shown in stick representation with the same colors as in Panel **a**. **d** Sequence alignment of MPXV A35R from clade II and clade I and its orthologs in VACV, VARV, and CPXV. The amino acids marked in blue contain mutations in A35R and its homologs across the abovementioned viruses. The amino acids highlighted in purple significantly interact with the amino acids in the BA345 Fab region. **e** Structural comparison of the binding modes of the MA42 Fab regions (left), MA49 (middle), and BA345 (right) with the A35R147–181 epitope. The Fab fragments are represented as molecular surfaces, while the A35R147–181 epitope is shown in cartoon representation. Residues participating in hydrogen bonds are depicted as stick models.

To elucidate the structural basis of the broad-spectrum neutralization of BA345, we determined the high-resolution crystal structure of its complex with the A35R antigen. The crystal structure of BA345–A35R precisely aligns with the computational predictions, as evidenced by a root-mean-square deviation (RMSD) of 0.682 Å and a template modeling score (TM score) of 0.985 (Supplementary Fig. S9 and Table S4). The TM score of 0.985 signifies that the predicted structure is highly similar to the experimental structure, differing only minimally in overall topology, thus confirming the EnsembleFold framework’s precision in modeling this complex. Moreover, the crystal structure revealed a conserved binding mechanism shared with MA49, wherein a single Fab domain engages the A35R dimeric interface through an analogous paratope-epitope geometry (Fig. 4a; Supplementary Fig. S8c). As with the binding with MA49, the heavy chain of BA345 dominates this interaction, contributing a total BSA of 1397.9 Å^2^, distributed between subunits A (809.9 Å^2^) and B (588.0 Å^2^). The light chain further contributes a 499.4 Å^2^ BSA, resulting in a total interface area of 1897.3 Å^2^, significantly exceeding that of the MA49–A35R complex (Fig. 4b; Supplementary Table S5). This larger interface area is stabilized by extensive molecular interactions, including hydrogen bonds, salt bridges, and hydrophobic contacts, as detailed below.

BA345 shares a similar interaction mechanism with MA49, wherein the HCDR3 region of the antibody is centrally positioned within the groove of the A35R dimer (Supplementary Fig. S8f). The extensive interactions with both the A and B subunits of A35R form a robust primary interaction interface. A network comprising multiple hydrogen bonds was identified between the BA345 heavy chain residues HFR1, HCDR1, HCDR2, HFR3, and HCDR3 and the residues in subunits A and B of A35R. The light chain improves binding between the two compounds through interactions between the LCDR1, LCDR2, and LCDR3 residues of BA345 and the corresponding residues in subunits A and B of A35R. Crucially, salt bridges were identified between HCDR2 residues R64 (subunit A: D170, E174) and HCDR3 residues R97 (subunit B: E174) (Fig. 4b, c; Supplementary Table S8). Finally, hydrophobic interactions involving both heavy and light chain residues further stabilized the ternary complex between BA345 and A35R. Comparative structural analyses of the MA42, MA49, and BA345 complexes demonstrated that BA345 binds to A35R with a larger interaction interface and increased topological complementarity.

As with MA42 and MA49, we assessed the conservation of the BA345 binding epitope on A35R across orthopoxviruses through sequence alignment. The results revealed that BA345 targets a set of highly conserved A35R residues (S114, Y116, E149, D150, K161, T162, Y166, D168, S169, D170, E174, V175, R176, and K177) across orthopoxviruses, including MPXV (clades I/II), vaccinia virus (WR strain), cowpox virus, and variola virus (Ind3/1967 isolate) (Fig. 4d; Supplementary Fig. S8d). The unique binding geometry of BA345, which leverages both framework and complementarity-determining region (CDR) residues, may create a more stable or adaptable binding interface. This characteristic enables BA345 to interact effectively with the conserved A35R residues shared across multiple orthopoxvirus species. By targeting these highly conserved epitopes, BA345 has the potential to maintain neutralizing activity against a broad range of orthopoxviruses, making it a promising candidate for development as a broad-spectrum therapeutic agent.

Notably, comparative structural analyses of the MA42–A35R, MA49–A35R, and BA345–A35R complexes revealed that residues 147–181 from A35R form a highly flexible loop that is critical for antibody recognition (Fig. 4e) and contributes the majority of the hydrogen bonds, salt bridges, and hydrophobic interactions across all three antibody complexes. Despite an S165T substitution in A35R of variola virus, the side chain properties of the antigen are maintained, preserving the structural integrity of the epitope. The universal conservation of this loop among orthopoxviruses highlights its potential as a key epitope, the neutralization of which can elicit robust immune responses. These findings provide a structural foundation for the rational design of vaccines targeting this conserved immunogenic loop within A35R.

### Broad-spectrum neutralization assessment of BA345 and MA49 against orthopoxviruses

**Fig. 5:**
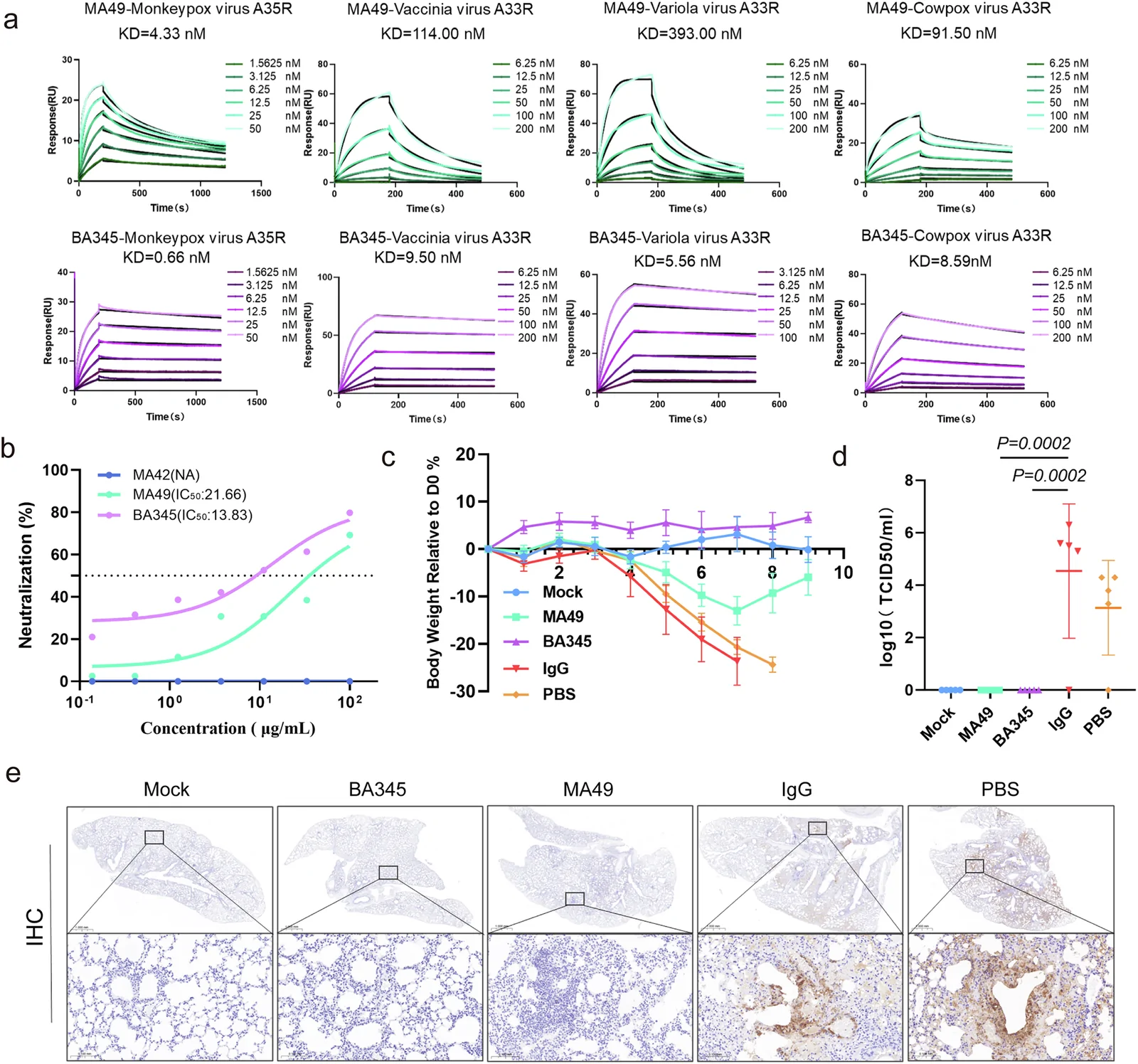
Broad-spectrum neutralization assessment of BA345 and MA49 against Orthopoxviruses. **a** Kinetics of the binding of MA49 and BA345 to the mpox virus A35R protein and the vaccinia virus, cowpox virus, and variola virus A33R proteins, as measured using surface plasmon resonance (SPR) assays (black = fitted curves). **b** Neutralizing potencies of MA42, MA49, and BA345 against the EEV form of vaccinia virus. **c** Body weight changes in BALB/c mice treated with BA345, MA49, IgG1, or PBS. **d** Viral titers in the lungs 8 days post-infection (dpi) as detected with a TCID50 assay. **e** Immunohistochemical (IHC) staining for VACV in the lung tissues of treated and control mice. Brown staining indicates positivity for the virus. The nuclei were counterstained with hematoxylin (blue). For the IHC images, the lower panel (scale bar = 0.100 mm) shows an enlarged view of the boxed area in the corresponding upper panel (scale bar = 1.000 mm).

Building on the structural insights into conserved epitopes, we next sought to functionally evaluate the broad-spectrum potential of BA345 and MA49 against representative orthopoxviruses. SPR analysis revealed that BA345 binds robustly to the A33R protein across species, with *K*_D_ values of 9.50 nM for vaccinia virus, 8.59 nM for cowpox virus, and 5.56 nM for variola virus. In contrast, MA49 showed more variable cross-reactivity with the A33R homologs, with *K*_D_ values of 114.00 nM for vaccinia, 91.50 nM for cowpox, and 393.00 nM for variola (Fig. 5a). The values for MA49 represent order-of-magnitude reductions in the binding efficiency for A33R relative to MPXV A35R (*K*_D_ = 4.33 nM), which are likely associated with the substitutions at residues 146 and 164. Next, the neutralization capacity of the two antibodies against the vaccinia virus was assessed with PRNT. BA345 exhibited effective activity (IC_50_ = 13.83 μg/mL), surpassing that of MA49 (IC_50_ = 21.66 μg/mL) (Fig. 5b). Thus, BA345 is a broad-spectrum neutralizing antibody with exceptional cross-genus potency, whereas MA49 demonstrates moderate activity that is limited to specific orthopoxviruses.

We then evaluated the protective efficacy of the mAbs BA345 and MA49 against lethal intranasal challenge with VACV (strain WR) in BALB/c mice. The mice were administered 10 mg/kg BA345, MA49, PBS, or an IgG1 isotype control antibody via intraperitoneal injection one day prior to virus challenge, followed by a second dose three days post-infection (dpi), in accordance with a previously reported protocol^33,34^. The baseline body weight of the BA345-treated mice was maintained throughout the 8-day observation period, whereas the MA49-treated mice exhibited transient weight loss but recovered to their initial weights by Day 8. In contrast, both the PBS- and IgG1-treated control groups displayed progressive weight loss, surpassing the 20% humane endpoint threshold by Day 7 (Fig. 5c). Viral titers in lung homogenates on Day 7 revealed nearly complete replication suppression in the BA345- and MA49-treated mice, with values at or below the assay detection limit, whereas the control groups presented high viral loads (Fig. 5d, e). Overall, BA345 achieved complete protection against a lethal VACV challenge, whereas MA49-based therapy resulted in significant but incomplete protection. The complete absence of replicating virus in BA345-treated animals, the previously demonstrated subnanomolar affinity of the mAb for variola A33R, and its low (single-digit) neutralization potency together reflect its translational potential as a panorthopoxvirus therapeutic agent.

### In vivo evaluation of the protective efficacy of BAL31/BA345 and BAL31/MA49 cocktails against Mpox virus infection

**Fig. 6:**
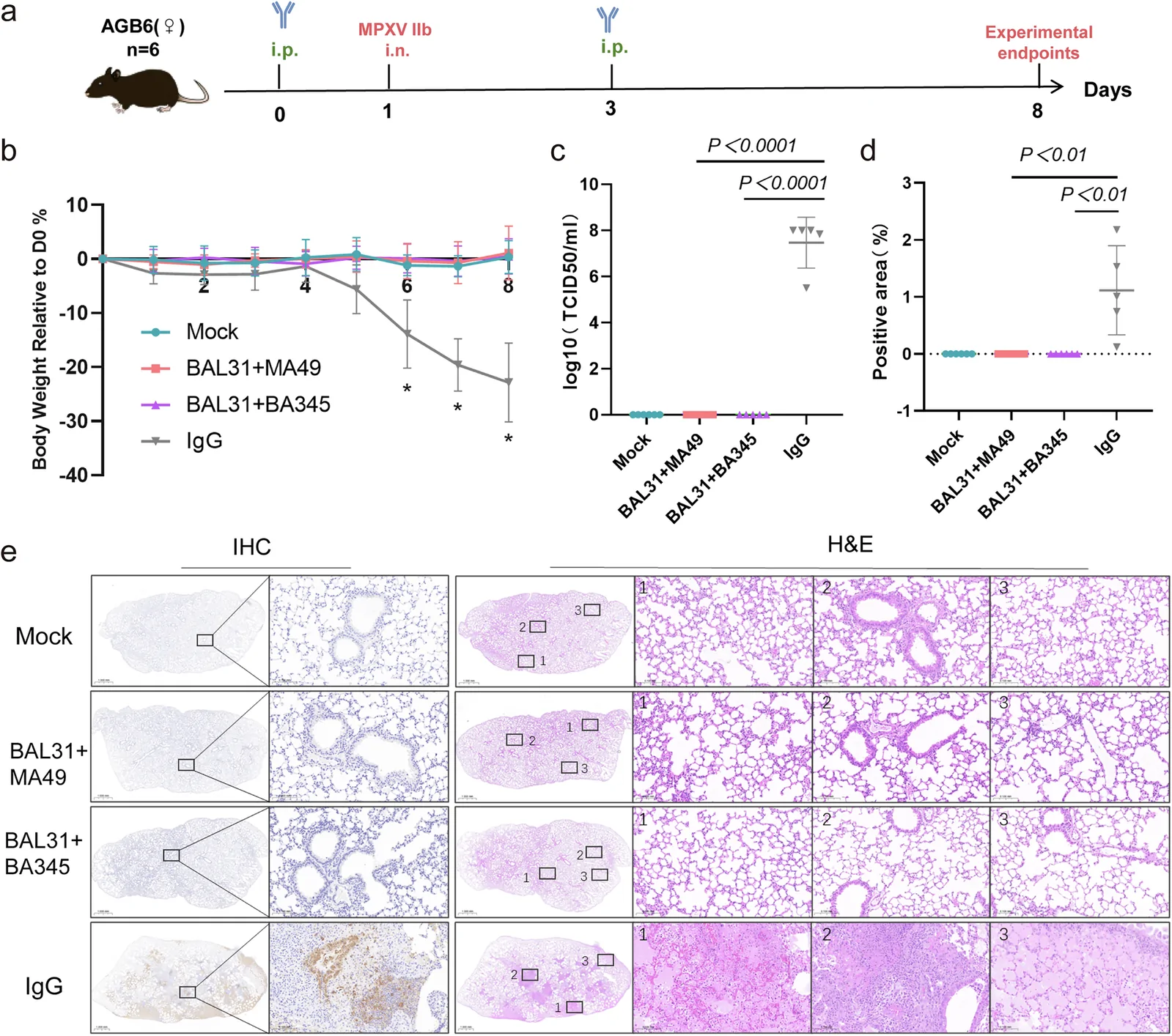
Protective efficacy of BAL31 + MA49/BAL31 + BA345 against MPXV clade IIb in vivo. **a** One day prior to virus challenge, female AGB6 (Ifngr1/Ifnar1-KO) mice aged 9–12 weeks were injected intraperitoneally (i.p.) with 200 µg/200 µL of one of the antibody cocktails or control reagent (*n* = 6 per group). The mice were subsequently deeply anesthetized with tribromoethanol and then injected intranasally (i.n.) with 4 × 10^5^ PFU/50 µL of the MPXV clade IIb strain. The mice were again injected with the drug cocktail or control reagent two days after exposure to the virus. **b** Body weight changes in the AGB6 mice. **c** Viral titers in the lungs 7 dpi as detected by TCID50 assays and qPCR. **d** Statistical analysis of the positive staining area ratio of MPXV-A29 by IHC. **e** IHC detection of the MPXV antigen in the lungs (MPXV-A29 viral protein) and hematoxylin and eosin (H&E) staining-based detection of pathological changes in the lungs. In the IgG group, “1” indicates severe bleeding between the alveoli, “2” indicates significant and extensive inflammatory cell infiltration in and around the bronchus, and “3” indicates significant and extensive interalveolar exudation of tissue fluid. For the IHC images, the right column (scale bar = 0.100 mm) is the enlarged area in the square box of the images in the left column (scale bar = 1.000 mm).

The pleomorphic nature of MPXV infection presents unique therapeutic challenges, as demonstrated by the distinct pathogenic mechanisms of its two virion morphotypes, EEV and IMV. While the EEV form achieves interhost transmission through an immune-evasive surface architecture, the IMV form achieves cell-to-cell dissemination through robust membrane fusion machinery^56,57^. On the basis of preliminary animal assays using vaccinia virus, we developed an antibody cocktail combining IMV-specific (BAL31) and EEV-neutralizing (BA345, MA49) antibodies. ELISA and structural modeling of the BAL31 Fab–A29L complex demonstrated that BAL31 mediates neutralization via all three CDRs, engaging a solvent‑exposed N‑terminal loop motif (residues 1–24) (Supplementary Fig. S10). In this subsection, AGB6 mice were randomly assigned to four treatment groups at the outset: two antibody‑cocktail groups (BAL31 + BA345 and BAL31 + MA49), an IgG1 isotype control group, and a mock control group. Therapeutic efficacy was then assessed in a mouse intranasal challenge model, with each cohort receiving 10 mg/kg of their assigned treatment via intraperitoneal injection before and after infection, combining prophylactic and therapeutic dosing to block viral entry and early replication. This prevention and treatment hybrid regimen more closely mimics clinical scenarios, such as postexposure emergency prophylaxis combined with early treatment. (Fig. 6a). While the IgG control animals exhibited progressive weight loss culminating in severe morbidity, the treated and mock cohorts maintained their baseline body masses throughout the observation period (Fig. 6b). TCID_50_ analysis revealed pulmonary viral loads below detection thresholds in the treatment and mock groups, whereas substantial viral titers were detected in the control group. Subsequent immunohistochemical (IHC) staining of the lung sections of the animals confirmed complete viral antigen clearance in the treated groups, in contrast with the control animals, whose lung samples demonstrated widespread antigen distribution (Fig. 6c, d). Further histopathological evaluation demonstrated marked attenuation of markers of pulmonary pathology in the treated animals, whereas the lungs of the control mice displayed extensive alveolar exudates, bronchiolar epithelial disorganization, perivascular leukocytic infiltration, and hemorrhagic necrosis, the samples from the antibody-treated groups presented only minimal peribronchial inflammation and an otherwise preserved parenchymal architecture (Fig. 6e). Collectively, these findings demonstrate that the antibody cocktail confers robust protection against MPXV infection through the coordinated neutralization of both EEV and IMV, coupled with the mitigation of infection-associated immunopathology. Critically, this dual-targeting strategy is anticipated to impose significant evolutionary constraints on MPXV, as the requirement for concurrent mutations is statistically less probable and often incurs substantial fitness costs, thereby potentially restricting viral dissemination and extending therapeutic durability.

## Discussion

The resurgence of Clade Ib MPXV, with its increased transmissibility and lethality, poses a significant threat to global health and necessitates the development of effective therapeutics. This study aimed to address this urgent need by identifying neutralizing antibodies, elucidating their mechanism of action, and developing antibody cocktail therapies. In this study, a computational-experimental platform was developed that systematically identified 5 fully human neutralizing antibodies against MPXV IMV and EEV surface antigens, with the elite candidate BA345 against A35R exhibiting superior neutralization potency across orthopoxviruses. The high-resolution X-ray structure of MPXV A35R in complex with neutralizing antibodies was determined, revealing a mechanism whereby these antibodies recognize and bind with a hyperconserved epitope of the A35R dimer to block host-cell entry. Building on these insights, we developed antibody cocktail therapies (BA345/BAL31 and MA49/BAL31) that exhibited effective therapeutic efficacy in murine MPXV challenge models, characterized by the marked attenuation of pulmonary histopathological lesions and increased viral clearance in respiratory tissues.

Deep learning frameworks have substantially improved the accuracy of protein–protein complex structure prediction, enabling high-throughput, structure-guided candidate selection that streamlines antibody discovery prior to extensive experimental validation. In this study, we established a platform that integrates scBCR-seq with EnsembleFold, a deep learning-based structure prediction method. From an initial repertoire of 21,646 paired antibody sequences, we first applied bioinformatic filters to select 196 candidate antibodies. EnsembleFold was subsequently used to predict the structures of the complexes with the target antigens (A35R, B6R, A29L, and H3L) and to rank the candidates on the basis of the quality of the predicted binding interfaces. The top 40 antibodies were chosen for experimental characterization, which revealed 12 fully human antibodies against MPXV EEV and IMV surface antigens, five of which exhibited significant neutralizing activity. Among these candidates, the lead candidates BA345 and MA49, both targeting A35R, demonstrated superior neutralization potency. While these results underscore the considerable potential of EnsembleFold for high-throughput antibody screening and accurate structural characterization, the limitations of this approach should also be acknowledged. Only a subset of the 40 computationally prioritized candidates were experimentally confirmed to exhibit binding activity, indicating that deep learning-based models still face challenges in fully capturing the complexity of certain antigen‒antibody interfaces, CDR loop dynamics, and the contributions of framework regions. Thus, although computational prescreening serves as a powerful enrichment strategy, it does not yet replace the need for thorough empirical validation; continued advances in modeling architectures and training datasets are expected to further improve predictive accuracy and experimental success rates.

Recent advances in structural biology have revolutionized our understanding of viral mechanisms and accelerated the development of antiviral therapies^19,45,48,58^. The MPXV A35R protein has emerged as a critically important target for therapeutic antibody development, as substantiated by our work and by multiple independent studies^42,43^. Our elite antibodies, BA345 and MA49, along with the recently reported EV35 series^42^ and mAbs 975/981^43^, all recognize the conserved groove of the A35R homodimer but exhibit distinct binding modes characterized by variable approach angles and divergent CDR loop conformations. This convergent yet structurally diverse recognition underscores the functional importance of the A35R dimer interface as a key site of vulnerability. Notably, effective A35R-directed antibodies have been isolated from individuals with different immunization backgrounds, including those receiving smallpox vaccines or recovering from MPXV infection. This consistency across immune histories suggests that structural or functional constraints may contribute to the conservation of the A35R groove, rendering it a preferential target of human humoral immunity. Collectively, these findings support the identification of A35R as a rationally grounded target worthy of further investigation for the development of orthopoxvirus countermeasures.

Despite this common epitope targeting, BA345 is distinguished by its discovery via a novel AI-driven platform, exceptional biophysical properties, and validated therapeutic potential. Structurally, among all reported A35R-targeting antibodies, BA345 has the largest buried surface area (1897.3 Å^2^), surpassing those of EV35-2 (1272 Å^2^), mAb 975 (1497.4 Å^2^), and mAb 981 (1332.7 Å^2^). Furthermore, compared with the 26 hydrogen bonds/5 salt bridges for EV35-2, 14 hydrogen bonds/2 salt bridges for mAb 975, and 11 hydrogen bonds/3 salt bridges for mAb 981, BA345 engages A35R through a more extensive network of noncovalent interactions (Supplementary Tables S9, S10). This binding interface underlies the subnanomolar affinity of BA345. Specifically, unlike other antibodies, BA345 recognizes a conserved epitope across orthopoxviruses, enabling the effective neutralization not only of MPXV and VACV but also significant cross-reactivity against the A33R homologs of cowpox and variola viruses, which demonstrated outstanding broad-spectrum potential. Importantly, the properties of BA345 are not only of mechanistic interest but also of therapeutic relevance. Compared with previously reported A35-targeting antibodies, BA345 achieved a deeper and more complete level of in vivo protection. Fantin et al. elegantly demonstrated that EV35-2, EV35-6, and EV35-7 effectively restricted VACV and MPXV dissemination and markedly improved survival in murine challenge models, with EV35-6 and EV35-7 even conferring complete survival against MPXV challenge. Notably, in those studies, protected animals still experienced considerable acute body weight loss, and EV35-2 provided only partial protection (75% survival), illustrating the clonal variability in protective efficacy among A35-directed antibodies^42^. Similarly, Ju et al. reported that mAbs 975 and 981 significantly reduced viremia, tissue viral burden, and pathological lesions in CAST/EiJ mice, yielding pronounced attenuation of disease severity^43^. Extending these important findings, BA345 conferred near-complete protection against lethal VACV challenge, maintaining the baseline body weight throughout the experiment and reducing pulmonary viral titers to below the detection limit. Furthermore, when combined with the IMV-targeting antibody BAL31, the BA345-based cocktail achieved stable weight maintenance and efficient viral clearance in the MPXV challenge model. This dual-targeting strategy simultaneously blocks EEV-mediated dissemination and IMV-mediated entry. Therefore, the added value of BA345 lies not only in its high-affinity recognition of a conserved A33R/A35 epitope and broad orthopoxvirus cross-reactivity but also in its capacity to elicit a near-asymptomatic protective phenotype in vivo, reinforcing its promise as a key component for next-generation orthopoxvirus antibody therapeutics.

In summary, our deep learning-based platform allows the efficient isolation and validation of elite antibodies through the integration of single-cell transcriptomics with deep learning-based structural prediction. We resolved the structure of MPXV A35R-antibody complexes and decoded conserved orthopoxvirus vulnerabilities, potentially guiding the development of panpoxvirus therapeutic strategies. Finally, dual antigen-targeting antibody cocktails demonstrate effective therapeutic efficacy in murine MPXV challenge models, exemplifying how the fusion of biotechnology and information technology could drive next-generation pandemic defenses by facilitating precision molecular engineering.

## Materials and methods

### Sources of patients

Five convalescent patients with monkeypox virus infection were recruited from the Second People’s Hospital of Tianjin, China. Peripheral B cells from these volunteers were isolated and enriched for subsequent analysis. Sample collection, processing, and laboratory procedures were approved by the Ethics Committee of the Second People’s Hospital of Tianjin (Approval No. Jin-ER-2023-76 Tianjin Second People’s Hospital Ethical Review). Written informed consent was obtained from each enrolled patient in accordance with the Declaration of Helsinki.

### Flow cytometry sorting of CD19^+^CD27^+^ B cells

Peripheral blood samples were obtained from patients at the Second People’s Hospital of Tianjin, China. PBMCs were isolated using the Ficoll density gradient centrifugation protocol (GE Healthcare). After isolation, erythrocytes were lysed with ACK lysis buffer (Life Sciences), and the remaining cells were passed through a 40 μm cell strainer to generate single-cell suspensions for further analysis. For surface marker staining, the cells were incubated on ice for 30 min with fluorochrome-conjugated mAbs in PBS supplemented with 1% bovine serum albumin (BSA). To reduce nonspecific binding, cells were pretreated with an anti-human Fc receptor-blocking antibody (Cat# 14-9161-73; Invitrogen). LIVE/DEAD Fixable Viability Dye (FVS780; Cat# 565388; BD Biosciences) was used to exclude dead cells. The following fluorochrome-conjugated antibodies were used for staining: Brilliant Violet 650™ anti-human CD27 antibody (Cat# 302828; BioLegend) and APC Mouse Anti-Human CD19 antibody (Cat# 555415; BD Biosciences). Stained cells were analyzed and sorted using flow cytometry to isolate the CD19^+^CD27^+^ B-cell population, which was used for subsequent experiments.

### scRNA-seq

Single-cell suspensions were prepared following the 10× Genomics® Cell Preparation Guide, optimizing the cell washing, counting, and concentration for abundant and limited samples. The cell suspensions were loaded onto Chromium microfluidic chips and processed using a Chromium Controller with Single-Cell 5’ v3 chemistry, depending on the project. Following the manufacturer’s instructions, reverse transcription and library preparation were performed using Chromium Single-Cell 3’ reagent kits. Sequencing was conducted on an Illumina platform. Raw sequencing data in FASTQ format were processed with the Cell Ranger pipeline (version: cellranger-9.0.1), which performed demultiplexing, alignment to the reference genome, and the generation of feature-barcode matrices. When additional quality control was needed, the raw reads were preprocessed using Trimmomatic to remove low-quality reads, trailing bases, adapter sequences, and short reads. The resulting clean reads were reevaluated with fastp to ensure high-quality data for downstream analyses.

### Generation and analysis of single-cell transcriptomes

Digital gene expression matrices were generated using the Cell Ranger pipeline, with unique molecular identifiers assigned to each gene and cell barcode. For multisample studies, the Cell Ranger aggr pipeline was used to aggregate data across samples, normalize sequencing depth, and recompute feature-barcode matrices. Seurat was used for further data processing and secondary quality control, with genes expressed in ≥ 3 cells and cells with ≥ 200 expressed genes retained.

Dimensionality reduction, clustering, and differential expression analyses were performed using Seurat and Cell Ranger reanalyses. Seurat’s canonical correlation analysis (CCA) was used to integrate the datasets, with the clustering resolution set to 0.6. Between-sample differential expression analysis was conducted using the edgeR package. The functional enrichment of marker genes was carried out with cluster profile for Gene Ontology (GO) and Kyoto Encyclopedia of Genes and Genomes (KEGG) pathway analyses, while Reactome pathway analysis was performed using ReactomePA. Protein‒protein interaction networks were constructed using the STRINGdb package to explore interactions between marker genes.

### Analysis of scBCR-seq

The BCR contig sequences were assembled and annotated using the “vdj” function of Cellranger (v7.1.0) with the GRCh38 reference genome^19,59^. Only contigs labeled as high confidence and productive were retained for clonotype analysis. The cells selected for further analysis required at least one heavy chain (IGH) and one light chain (either IGL or IGK). For cells with two or more assembled IGHs or IGLs/IGKs, the dominant heavy or light chain was identified as the IGH or IGL/IGK sequence with the highest UMI count. Each unique IGH-IGL/IGK pair was regarded as a clonotype, and the degree of clonality of a given clonotype was assessed by the frequency of B cells sharing that clonotype. SHM was calculated as the total number of mismatches and gaps divided by the length of the heavy-chain VDJ DNA sequence. Mismatches and gaps were identified by aligning the heavy-chain VDJ DNA sequence to the germline sequence (sourced from the international ImMunoGeneTics information system (IMGT)) using Igblast (v1.22.0)^60^.

To identify high-confidence clonotypes for downstream characterization, we initially applied the following criteria: a clonotype frequency ≥ 1, the use of the IGHG1 isotype, the presence of at least one MBC, and an SHM rate > 2%, indicating affinity maturation. This stringent filtering, supported by prior studies¹⁹, aimed to enrich for clonotypes representing bona fide antigen-experienced B cells. However, because relatively few clonotypes satisfied the > 2% SHM threshold in our dataset, we further incorporated CDR3 length and sequence feature analyses to broaden the candidate pool and increase the likelihood of capturing neutralizing antibodies. In addition, select antibodies against IGHG2, IGHG3, and high-frequency IGHM isotypes were included for exploratory testing. From the initial 21,646 productive V–J pairings, this multistep selection yielded 196 candidate antibody sequences for downstream characterization.

### Antibody–antigen complex structure prediction

EnsembleFold is a deep learning framework for predicting the structures of antibody‒antigen complexes. Building on our CASP15 champion DMFold pipeline^11^, EnsembleFold introduces two key advances: (1) an updated DeepMSA^61^ pipeline, which integrates substantially expanded databases to construct multiple sequence alignments (MSAs), and (2) an enhanced structure-sampling engine, which leverages a modified AlphaFold3^62^ structure module to generate diverse and accurate structural models.

In the MSA construction step, DeepMSA2 is used to generate hiWghly informative MSAs to improve protein structure prediction. First, starting from the input sequences, the DeepMSA2-Monomer pipeline generates up to ten MSAs via three parallel blocks (dMSA, qMSA, and mMSA) by leveraging a comprehensive set of genomic and metagenomic sequence databases, comprising more than 40 billion sequences. The three MSA generation blocks integrate sequence search tools, including HHblits^63^, Jackhmmer and HMM^64^, and each block follows a similar multistage process in which the initial query is searched against a sequence database. If a sufficient number of effective sequences is not obtained in the early stages (usually for antigens), iterative searches into larger databases are attempted. Second, for antibody‒antigen complexes, the top M MSAs from each of theN chains (the antigens, light chain, and heavy chain of the antibodies) are paired to form ${M}^{N}$ hybrid alignments, with M adjusted for computational efficiency. Third, the paired monomeric MSAs are grouped according to UniProt species and ordered by sequence identity relative to the query sequences. Orthologous sequences from the same species are concatenated to create composite multimeric MSAs, with gaps inserted to account for any missing chain segments. Finally, the top K (K is determined on the basis of whether it is involved in a screening task or structural modeling task) and multimeric MSAs for antibody‒antigen complexes are selected on the basis of a combined scoring approach considering MSA depth and predicted local distance difference test (pLDDT) scores.

In the model generation step, EnsembleFold employs a modified AlphaFold3 modeling engine. The MSAs from the first step serve as input features for this modeling engine. Key modifications to the AlphaFold3 modeling engine include (i) using templates or not, (ii) adjusting the dropout rate, (iii) generating a higher number of decoys than the default setting (five models), (iv) applying or omitting the early stop strategy, and (v) increasing or decreasing the number of modeling iterations. The final models are ranked on the basis of the QA score, which is a linear combination of the pTM and the ipTM. A higher QA score indicates greater confidence in the predicted structure and its intermolecular interface.

$${QA}=0.8\times {iptm}+0.2\times {ptm}$$

### Virtual screening of binding antibodies against the monkeypox virus

The necessary ability for a neutralizing antibody is its ability to stably bind to the target antigen and form a durable complex. To efficiently identify such antibodies, we employed EnsembleFold to predict antibody‒antigen complex structures and screened candidate antibodies likely to form stable complexes with specific MPXV antigens. These computationally selected antibodies were then prioritized for subsequent ELISA binding assays, thereby significantly improving the efficiency of the screening process.

The core principle of this strategy is that EnsembleFold can effectively distinguish binders from nonbinders on the basis of structural-modeling confidence scores. Specifically, we performed in silico modeling of 196 candidate antibodies in complex with four target antigens: A35R, B6R, A29L, and H3L. Given the dimeric nature of A35R, we considered two binding configurations: one antibody per dimer and two antibodies per dimer. For the other three monomeric antigens, only one antibody copy was adopted when modeling the complex. For each of the top 5 MSA combinations, we generated 20 random seeds, and five structural models were produced for each seed. The number of recycling iterations per model was set to the default value of ten. In total, this yielded 500 structural models per antibody–antigen complex. The model confidence was assessed using the QA score. The highest-scoring model for each antibody–antigen pair was selected as its representative structure. The top models were then grouped by antigens and ranked according to their QA scores. For each of the four antigens, 40 antibody candidates associated with the highest-ranking complexes were selected for ELISA binding assays.

### Cell lines, plasmids, and viruses

Hi5 (*Trichoplusia ni*) and Sf9 (*Spodoptera frugiperda*) cells were purchased from Union Biotech (Shanghai) and cultured in ESF921 Expression Medium (Expression Systems). The 293-F cells were purchased from Gibco and cultured in FreeStyle 293 Expression Medium (Gibco). Vero E6 cells (Priscilla) were inoculated in DMEM supplemented with 10% guinea pig complement (Cat# S4990; Solarbio). The codon-optimized wild-type cDNA of MPXV A35R (accession No. UUK29896.1), B6R (accession No. UUK29916.1), A29L (accession No. UUK29889.1), and H3L (accession No. UUK29844.1) was synthesized by Tsingke Biotechnology. The codon-optimized wild-type cDNAs of VACV A33R (accession No. YP_233038.1), Cowpox virus A33R (accession No. NP_619952.1), and Variola virus A33R (accession No. NP_042184.1) were synthesized by Tsingke Biotechnology. Monkeypox virus was isolated from a skin herpes swab from a clinical case at Guangzhou Eighth People’s Hospital (MPXV clade IIb: hMpxV/China/GD-GZ8H-0613-5/2023; GISAID accession ID: EPI ISL 18040906). Vaccinia virus WR was obtained from Professor Fang Minmin of Henan University.

### Protein expression and purification

The coding sequences for MPXV A35R (98–182) and B6R were cloned and inserted into the pHBM-8His expression vector, which includes an N-terminal His-tag for affinity purification. The recombinant plasmids were transfected into Sf9 insect cells using Cellfection II Reagent (Invitrogen) according to the manufacturer’s instructions. Seven days post-transfection, the P0 virus was harvested and subsequently amplified to generate high-titer viral stocks. For recombinant protein expression, Hi5 cells were cultured at a density of 2 × 10^6^ cells/mL and infected with high-titer baculovirus at a multiplicity of infection (MOI) of 5. The culture supernatant was collected 72 h post-infection by centrifugation at 4000 × *g* for 20 min at 4 °C to remove cell debris. Purification of the recombinant proteins was performed in two steps. First, the supernatant was applied to Ni-NTA resin (GE Healthcare) under native conditions for affinity purification. The eluted proteins were then subjected to size-exclusion chromatography (SEC) using a Superdex 200 Increase 10/300 GL column (GE Healthcare) preequilibrated with buffer containing 50 mM Tris and 200 mM NaCl (pH 8.0). This two-step purification process yielded highly pure target proteins suitable for structural and functional analyses.

The A29L and H3L genes of MPXV were cloned and inserted into the pET-15b expression vector, which includes an N-terminal His-tag for affinity purification. The recombinant plasmids were subsequently transformed into BL21 (DE3) *Escherichia coli* cells for protein expression. Transformed cells were initially cultured at 37 °C with shaking (200–220 rpm) until the optical density at 600 nm (OD600) reached 0.6–0.8. Protein expression was induced by adding isopropyl β-D-1-thiogalactopyranoside (IPTG) to a final concentration of 500 μM, followed by incubation at 16 °C for 16 h. The cells were then harvested by centrifugation at 4 °C and 6000 × *g* for 15 min. For protein purification, the cell pellets were resuspended in lysis buffer (50 mM Tris-HCl, 200 mM NaCl, pH 8.0) and lysed by sonication on ice. The lysate was clarified by centrifugation at 12,000 × *g* for 30 min at 4 °C. Recombinant proteins were first purified from the soluble fraction using Ni-NTA affinity chromatography (GE Healthcare) under native conditions. Eluted proteins were further purified by size-exclusion chromatography using a Superdex 200 Increase 10/300 GL column (GE Healthcare) preequilibrated with buffer containing 50 mM Tris-HCl and 300 mM NaCl (pH 8.0). Protein purity was assessed by SDS‒PAGE.

The A33R (98–186) proteins from VACV, cowpox virus, and variola virus were expressed using the 293 F mammalian cell expression system. The recombinant plasmids were combined with polyethyleneimine (PEI) transfection reagent and incubated at room temperature for 25 min to allow the formation of DNA‒PEI complexes. These complexes were subsequently added to 293F cells at a density of 1 × 10⁶ cells/mL in suspension culture. The cells were maintained in a humidified incubator at 37 °C with 5% CO₂ and shaking at 120 rpm. Four days after transfection, the culture supernatant was collected by centrifugation to remove cells and cellular debris. The clarified supernatant was subjected to initial purification of recombinant A33R proteins using Ni-NTA affinity chromatography (GE Healthcare) under native conditions. The eluted proteins were collected for further purification and subsequent downstream applications.

### Antibody production and Fab fragment generation

Heavy and light chain plasmids (196 pairs in total) were cotransfected into HEK293 cells using TF02 transfection reagent (Cat# STF02; Sinofection) according to the manufacturer’s protocol. The cells were maintained in SMS293-SUPI medium (Cat# M293-SUPI-100; Sinofection) at 37 °C in a humidified incubator with 5% CO₂. Four days post-transfection, aliquots of the culture supernatant were harvested for preliminary binding analysis via ELISA.

Six days post-transfection, the culture supernatant was collected, and mAbs were purified using a HiTrap Protein A HP column (GE Healthcare) under native conditions. The purified antibodies were used to prepare Fab fragments by enzymatic digestion with papain. Briefly, the antibodies were incubated with papain at 37 °C for 8 h in digestion buffer optimized for proteolysis. The reaction mixture was then applied to a Protein A column to remove undigested IgG and Fc fragments. The Fab fragments were eluted, collected, and dialyzed against PBS to remove residual buffer components and prepare the samples for downstream applications.

### ELISA binding assay

To evaluate antibody binding, microplates were coated overnight at 4 °C with 2 μg/mL recombinant proteins, including MPXV A35R, B6R, H3L, A29L, and A33R, along with proteins derived from VACV, Variola virus, and Cowpox virus, which were subsequently diluted in PBS. After coating, the plates were washed three times with PBS containing 0.05% Tween-20 (PBST) and blocked with 5% nonfat milk in PBST for 1 h at room temperature.

After the blocking step, 100 μL of mAb at a concentration of 1 μg/mL was added to each well and incubated at 25 °C for 1 h. The plates were then washed three times with PBST, and 100 μL of horseradish peroxidase (HRP)-conjugated anti-human IgG (H + L) secondary antibody (1:5000 dilution; Cat# 109-035-098; Jackson ImmunoResearch) was added. After an additional 1-h incubation at 25 °C, the plates were thoroughly washed with PBST.

For detection, 100 μL of TMB substrate solution (Cat# PR1200; Solarbio) was added to each well, and the enzymatic reaction was allowed to proceed for 15 min at room temperature in the dark. The reaction was terminated by adding 50 μL of stop solution (Cat# C1058; Solarbio) to each well. The absorbance was measured immediately at 450 nm using a microplate reader.

### Binding epitope prediction for A35R-neutralizing antibody complexes

Following ELISA screening, 12 antibodies that exhibited strong binding signals (OD450 ≥ 2) were identified as candidates for further structural modeling. These included MA12, MA42, MA49, BA345, and BA382, which target the A35R antigen; BB07, BB207, and BA34, which target B6R; BAL31 and MAL25, which target A29L; and HB05 and HB32, which target H3L. These antibody‒antigen complexes were subjected to more refined structure predictions using EnsembleFold. In contrast to the initial virtual screening step, all available multimeric MSAs were used for each complex. For each MSA combination, five random seeds were generated, and two structural models were produced per seed. The number of recycling iterations per model was reduced to seven to promote extensive conformational sampling. This modeling strategy yielded tens of thousands of structural models per antibody–antigen complex. These models were then ranked by their QA scores, and the top-ranked model for each complex was selected for downstream analysis. Epitope binding was performed on the highest-confidence predictions.

### SPR

The binding kinetics and affinity of the mAbs for A35R/B6R/A29L/H3L were analyzed by SPR (Biacore 8 K, Cytiva). Specifically, purified A35R/B6R/A29L/H3L proteins were covalently immobilized on CM5 sensor chips via amine groups in 10 mM sodium acetate buffer (pH 5.0). By calculating the amount of coupling proteins, Rmax was less than 100. SPR assays were run at a flow rate of 30 mL/min in 1× HBS-EP buffer (Cat# BR100669; Cytiva). Purified mAbs were injected after serial dilution within an appropriate concentration range. The dilution factor was 1:4. The resulting data were fit to a bivalent analyte model using Biacore Insight Evaluation Software.

### EEV antibody neutralization assays

The day before the experiment, Vero E6 cells were inoculated in 96-well culture plates at a density of 1 × 10^4^ cells/well. A total of 30–50 PFU/100 μL of virus (MPXV clade IIb) was added to DMEM supplemented with 20% guinea pig complement (Cat# S4990; Solarbio) and 10 μg/mL IMV-closed antibody (anti-L1R-M12B9^65^) solution and incubated at 37 °C for 1 h. The antibodies were subsequently diluted 3-fold starting from 100 µg/mL and then mixed with an equal volume of the above virus-complement mixture, resulting in a final complement concentration of 10%. The antibody-virus mixture was incubated at 37 °C for 1 h and added to the wells to examine the cytopathic effect for 3 dpi. The absence of cytopathic effects in the well was defined as protection. The half-maximal inhibitory concentration (IC_50_) was calculated using GraphPad Prism software.

### IMV antibody neutralization assays

The day before the experiment, Vero E6 cells were inoculated in 24-well culture plates at a density of 1 × 10^5^ cells/well. MPXV clade IIb IMV (40–80 PFU/100 μL) was first mixed with guinea pig complement to obtain a 20% (v/v) complement concentration. This mixture was then combined with an equal volume of serially diluted antibodies (starting from 200 µg/mL), yielding a final complement concentration of 10%. After incubation at 37 °C for 1 h, 200 µL/well of the mixture was added to the cell monolayers, which were subsequently incubated for another 1 h at 37 °C. The inoculum was removed, and the cells were overlaid with 1 mL of 1.5% agar. The plates were incubated inverted for 3 days and then fixed with 1 mL of 0.1% crystal violet fixative overnight. After the agar overlay was removed, the plaques were counted, and the IC₅₀ was calculated using GraphPad Prism.

### Crystallization

The purified MA49/A35R and MA42/A35R fusion proteins were concentrated to 10 mg/mL and 20 mg/mL, respectively, and subjected to crystallization screening using the vapor-diffusion sitting-drop method at 16 °C. The screening kits used included Index, Crystal Screen, PEG/Ion, and Salt RX (Hampton Research) as well as Wizard I-IV (Emerald BioSystems). Crystals of the MA49/A35R complex appeared after seven days under conditions comprising 0.1 M sodium citrate tribasic dihydrate (pH 5.0) and 30% (v/v) polyethylene glycol monomethyl ether 550. Crystals of the MA42/A35R complex formed after three days under conditions of 0.1 M imidazole (pH 7.0) and 20% (v/v) Jeffamine® ED-2001 (pH 7.0). All the crystals were dehydrated and cryoprotected in a 30% (v/v) glycerol solution before being flash-cooled in a dry nitrogen stream at 100 K for X-ray diffraction data collection.

### X-ray data collection, processing, and structure determination

X-ray diffraction data were collected at the Shanghai Synchrotron Radiation Facility (SSRF) beamlines BL18U1 and BL19U1 at 100 K. The datasets were indexed, integrated, and scaled using the HKL2000 software suite. Initial phases for the BA345/A35R, MA49/A35R, and MA42/A35R complex structures were determined via molecular replacement with the PHENIX software suite. The preliminary models were manually adjusted to the experimental electron density maps using COOT^66^ and further refined with PHENIX^67^. The model geometry was validated using MolProbity, ensuring high-quality structural refinement. The final crystallographic data processing and refinement statistics are provided in Supplementary Tables S1–S3. Structural visualizations were generated using PyMOL (https://www.pymol.org) and UCSF ChimeraX (https://www.cgl.ucsf.edu/chimerax).

### Antibody‒antigen complex structure modeling quality assessment

The root-mean-square deviation (RMSD) and the template modeling score (TM score)^68^ were used to quantitatively assess the structural similarity between the predicted model and experimental structure in this work.

$${\rm{TM}}-{\rm{score}}=\max \left[\frac{1}{N}\mathop{\sum }\limits_{i=1}^{L}\frac{1}{1+{\left(\frac{{d}_{i}}{{d}_{0}}\right)}^{2}}\right]$$

where *N* represents the length of the experimental structure, *L* denotes the number of aligned residues, ${d}_{i}$ is the Euclidean distance between the *i*-th pair of aligned residues, and ${d}_{0}=1.24\times \sqrt[3]{N-15}-1.8$ is a distance scaling factor. The maximum is taken over all possible structural superpositions. The TM score ranges from 0 to 1, with higher values indicating a closer similarity to the correct topology. TM scores were calculated for the three A35R-targeting neutralizing antibodies with high-resolution X-ray crystal structures using US-align^69^.

### Prophylactic and therapeutic efficacy studies in mice

One day before the virus challenge, five female BALB/c mice aged 6–8 weeks per group were injected intraperitoneally (i.p.) with 200 μL of antibody or control reagent. The mice were deeply anesthetized with isoflurane and then injected intranasally (i.n.) with 5 × 10^4^ PFU/50 μL of the VACV WR strain. The mice were again injected with the drug on the second day after infection. Animals were observed daily for mortality and morbidity, body weights were measured, and lungs were harvested on Day 8 of infection for virus titration and histopathological examination. Animals that lost more than 25% of their initial body weight were euthanized by animal ethics protocols.

### Protective and therapeutic effects of the antibody cocktail in MPXV-infected mice

Female AGB6 mice (Ifngr1/Ifnar1-KO, 9–12 weeks old) were obtained from Shanghai Model Organisms Center, Inc. The animals were housed in individually ventilated cages (IVC) under biosafety level 3 (BSL-3) conditions (temperature: 22 ± 2 °C) with ≤ 6 mice per cage and provided with autoclaved water, feed, and bedding. All experimental procedures were approved by the Institutional Animal Care and Use Committee (IACUC) of the Institute of Infectious Diseases, Guangzhou Eighth People’s Hospital Affiliated with Guangzhou Medical University (Ethics Approval No. YHS-2024-10).

One day prior to the challenge, groups of six AGB6 mice were intraperitoneally administered 200 μL of antibodies or control reagents. The mice were deeply anesthetized with tribromoethanol (0.15 mL/10 g body weight) and intranasally inoculated with 4 × 10^5^ PFU of the MPXV clade IIb strain in 50 μL of PBS. A second dose was administered 48 h post-infection. Clinical symptoms and body weight were monitored daily. Lungs were harvested on Day 7 post-infection for viral titer quantification and histopathological analysis.

### Viral load measurement (TCID_50_)

One day ago, 96-well plates were prepared with 1 × 10^4^ cells per well. The tissue homogenate stored in 2% FBS DMEM was centrifuged, and the supernatant was diluted 10^−1^ to 10^−7^ and then added to 96-well plates, resulting in 6 wells per dilution. The cytopathic effect (CPE) was observed after 3 days, and the log(TCID_50_/mL) was calculated.

### H&E and IHC staining

The samples were first rinsed in xylene to remove paraffin. The slides were then rehydrated through increasing concentrations of alcohol and washed with phosphate-buffered saline. For H&E staining, lung tissue sections were stained with Gill’s hematoxylin and eosin Y. For IHC, the sections were sealed with 10% goat serum and incubated with a vaccinia virus antibody (Abcam: Ab35219) for 1 h, followed by secondary antibody for 30 min, followed by hematoxylin and DAB staining. After restaining with hematoxylin, the sections were dehydrated, coverslipped, and sealed.

### Ethics statements

The sample collection, processing, and laboratory procedures were approved by the Ethics Committee of the Second People’s Hospital of Tianjin (Approval No. Jin-ER-2023-76 (Tianjin Second People’s Hospital Ethical Review)). Informed consent to participate in the study was obtained from all participants. All experimental procedures were approved by the Institutional Animal Care and Use Committee (IACUC) of the Institute of Infectious Diseases, Guangzhou Eighth People’s Hospital Affiliated with Guangzhou Medical University (Ethics Approval No. YHS-2024-10).

### Statistical analyses

All the statistical analyses were conducted using GraphPad Prism 8.0.2 or OriginPro 2023b. Detailed information regarding the methods employed for calculating statistical significance can be found in the figure legends corresponding to each analysis. The number of experimental replicates (including replicate wells) or biological replicates (representing the number of subjects in each group) is documented within the relevant figure legends, results, or method sections. The determination of statistical significance is indicated in each figure legend.

## Supplementary information


Supplementary information


## Data Availability

Density maps and structure coordinates have been deposited into the Protein Data Bank (PDB) with accession codes 9UE8, 9UE0, and 9UDQ. Source data are provided with this paper.
