# Supplementary material for: Cross-protective human antibodies against the Mpox virus discovered through structure-guided screening
Source: Cell Discov. 2026 Sep 1;12:64. doi: 10.1038/s41421-026-00916-2 (PMC13534486; doi:10.1038/s41421-026-00916-2)
Supplement: Supplementary file 1 [file 41421_2026_916_MOESM1_ESM.pdf]

Supplementary Materials for

Cross-protective human antibodies against the Mpox virus discovered through structure-guided screening

Dongdong Sun[1,2,3,4$], Zihan Jia[1$], Hongyu Han[3,5$], Nan Zhang[1$], Yiying Guo[6$], Ruitian Hou[3], Wenli Zhao[1], Haiyan Yu[1], Lulu Wang[1], Yuhui Wang[1], Jun Dai[3], Hang Shang[1], Yamin Liu[7], Ding Li[1], Wenhui Tao[1], Zixian Sun[3], Ying Li[7*], Zhiyu Ni[8*], Wei Zheng[1,6*], Haisheng Yu[3*], Yu Guo[1,2,9*]

$ These authors contribute equally to this work: D.S., Z.J., H.H., N.Z., Y.Y.G.

*Correspondence should be sent to: Y.G (guoyu@nankai.edu.cn), H.Y(yuhaisheng@gzhmu.edu.cn) , W.Z(jlspzw@nankai.edu.cn), Z.N (nizhiyu@hbu.edu.cn) and Y.L(liying9886@126.com)

The PDF file includes:

Supplementary Fig. 1-10

Supplementary Table. 1-10

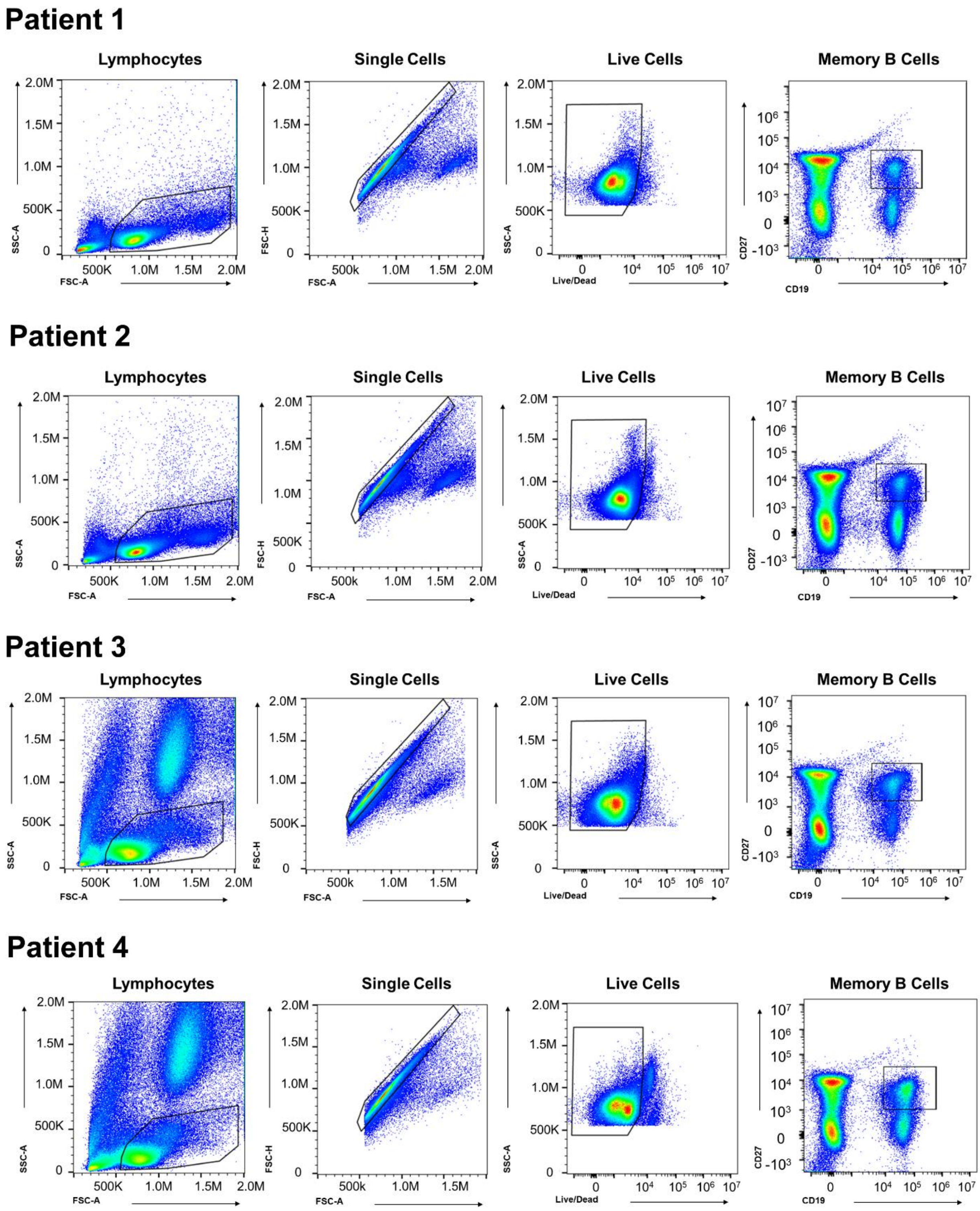

Supplementary Fig. 1 | Flow cytometry gating strategy for memory B cells in the PBMCs from the four patients.

Step 1: Gating for the primary population and removal of cell debris; Step 2: Exclusion of clumped or sticky cells to ensure that only single cells remain; Step 3: Removal of dead cells; Step 4: Gating for the $CD19^+CD27^+$ B cells.

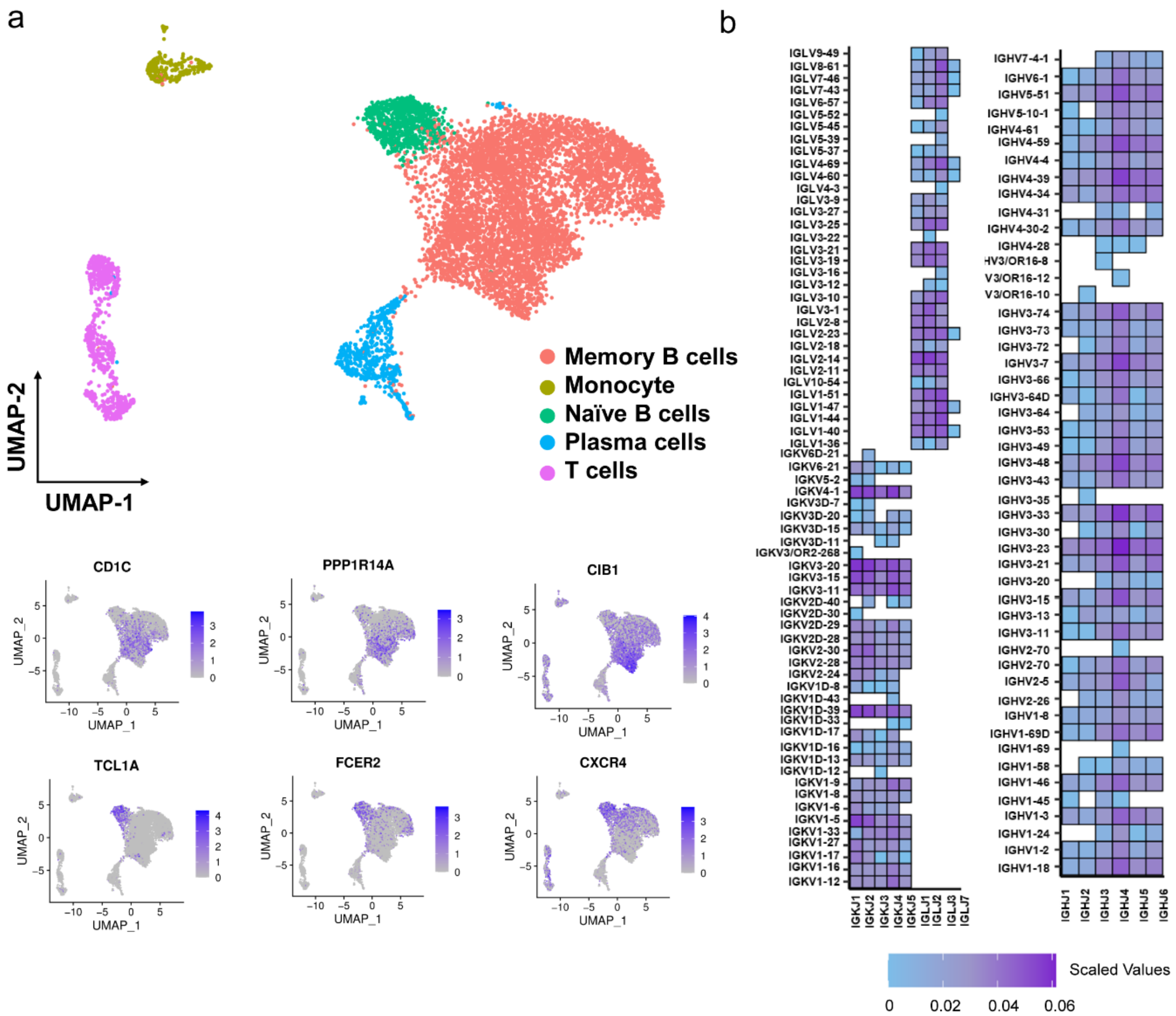

Supplementary Fig. 2 | UMAP plot of 6827 B cells from Group 2. a, Orange and green correspond to memory B cells and naïve B cells, which are characterized by high expression of CD1C/CIB1/PPP1R14A and TCL1A/FCER2/CXCR4, respectively. **b,** VDJ gene rearrangements for heavy chains and light chains of Group 2.

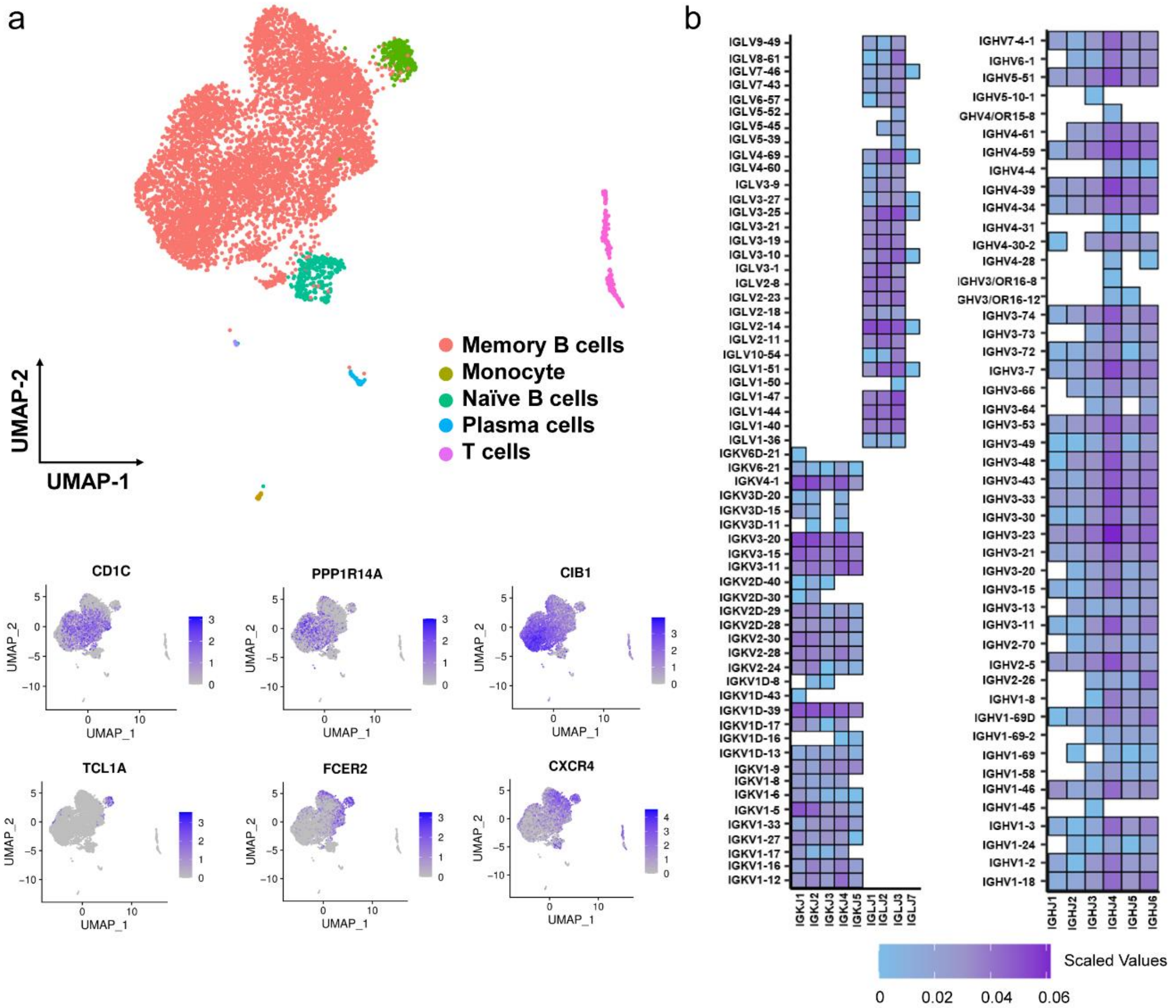

Supplementary Fig. 3 | UMAP plot of 7687 B cells from Group 3. a, Orange and green correspond to memory B cells and naïve B cells, which are characterized by high expression of CD1C/CIB1/PPP1R14A and TCL1A/FCER2/CXCR4, respectively. **b,** VDJ gene rearrangements for the heavy chains and light chains of Group 3.

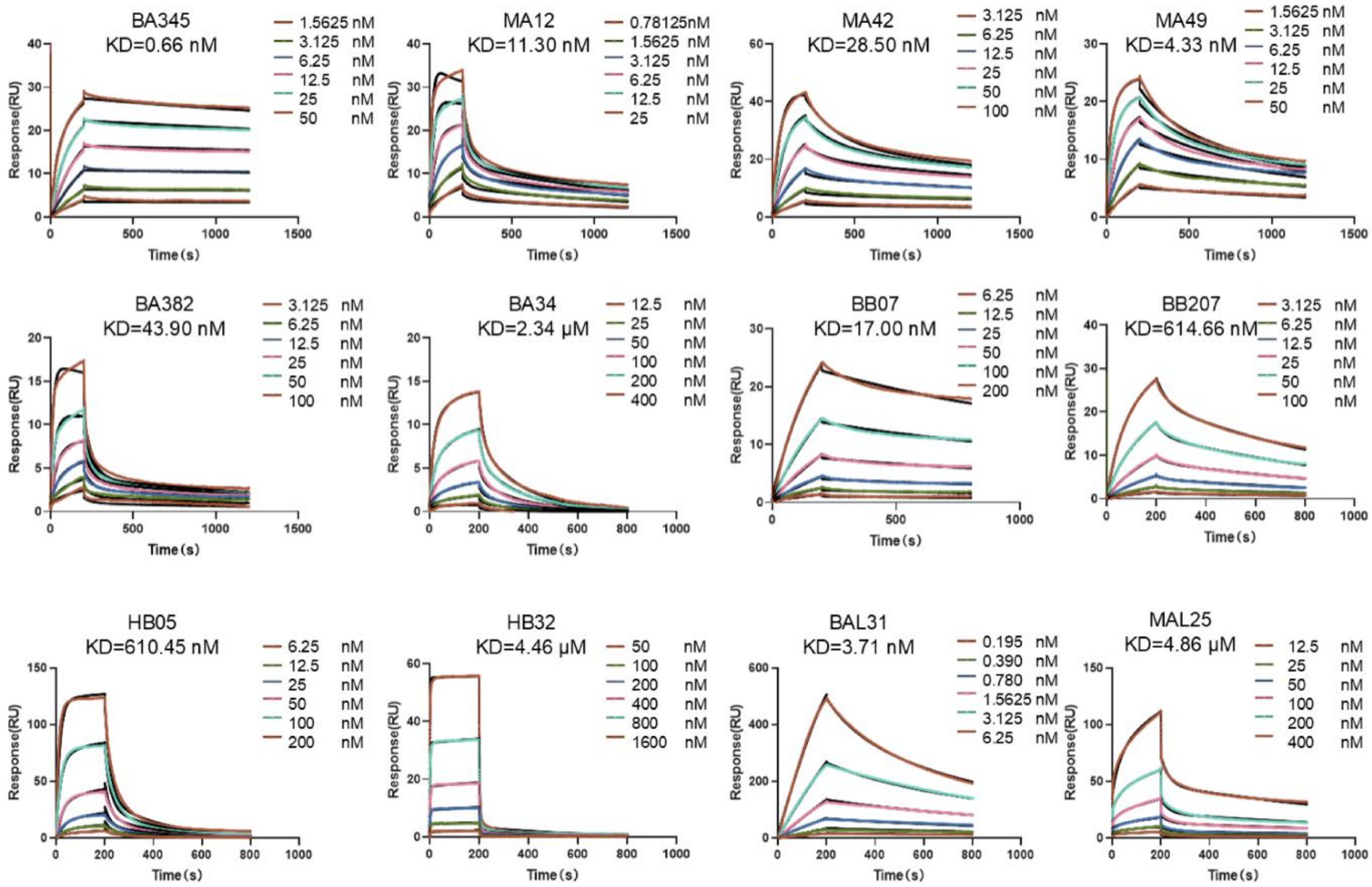

Supplementary Fig. 4 | Assessment of antibody-antigen binding affinity. Binding kinetics of MA12, MA42, MA49, BA345, and BA382 to the A35R protein; BB07, BB207, and BA34 to the B6R protein; BAL31 and MAL25 to the A29L protein; and HB05 and HB32 to the H3L protein, as measured using surface plasmon resonance (SPR) assays (black = fitted curves).

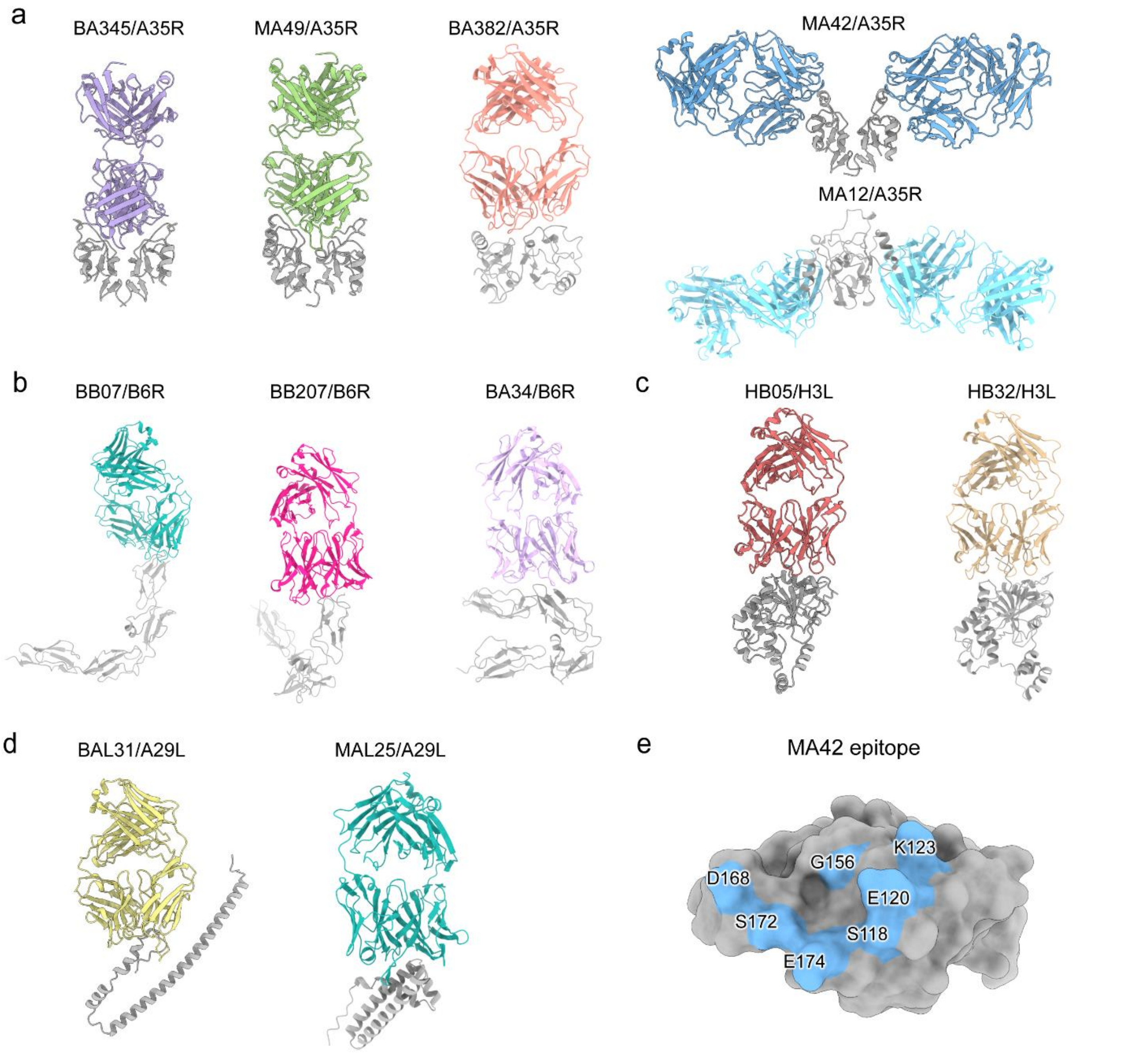

Supplementary Fig. 5 | Predicted structures of antigen-antibody complexes. a, Predicted complex structures of A35R with its antibodies BA345, MA49, BA382, MA42, and MA12. **b,** Predicted complex structures of B6R with its antibodies BB07, BB207, and BA34. **c,** Predicted complex structures of H3L with its antibodies HB05 and HB32. **d,** Predicted complex structures of A29L with its antibodies BAL31 and MAL25. **e,** Binding epitopes of the MA42-A35R complex as predicted by EnsembleFold.

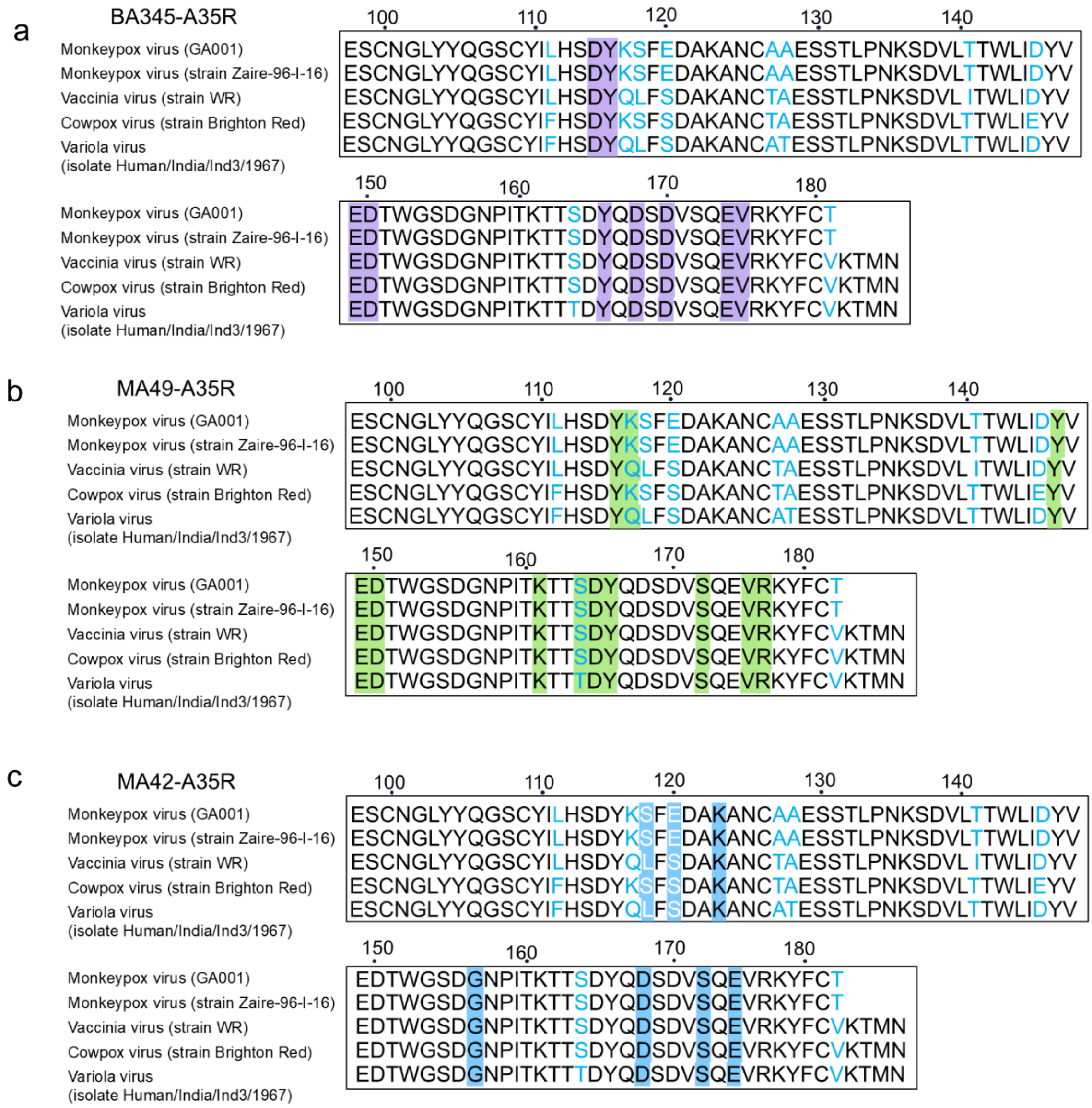

Supplementary Fig. 6 | Predicted binding epitopes of A35R recognized by BA345, MA49, and MA42. a, Sequence alignment of MPXV A35R from clade II and clade I and its orthologs in VACV, VARV, and CPXV. Predicted binding epitopes of A35R recognized by BA345 with A35R; the involved amino acids are highlighted in purple. **b,** Sequence alignment of MPXV A35R from clade II and clade I and its orthologs in VACV, VARV, and CPXV. Predicted binding epitopes of A35R recognized by MA49; the involved amino acids are highlighted in green. **c,** Sequence alignment of MPXV A35R from clade II and clade I and its orthologs in VACV, VARV, and CPXV. Predicted binding epitopes of A35R recognized by MA42; the involved amino acids are highlighted in blue.

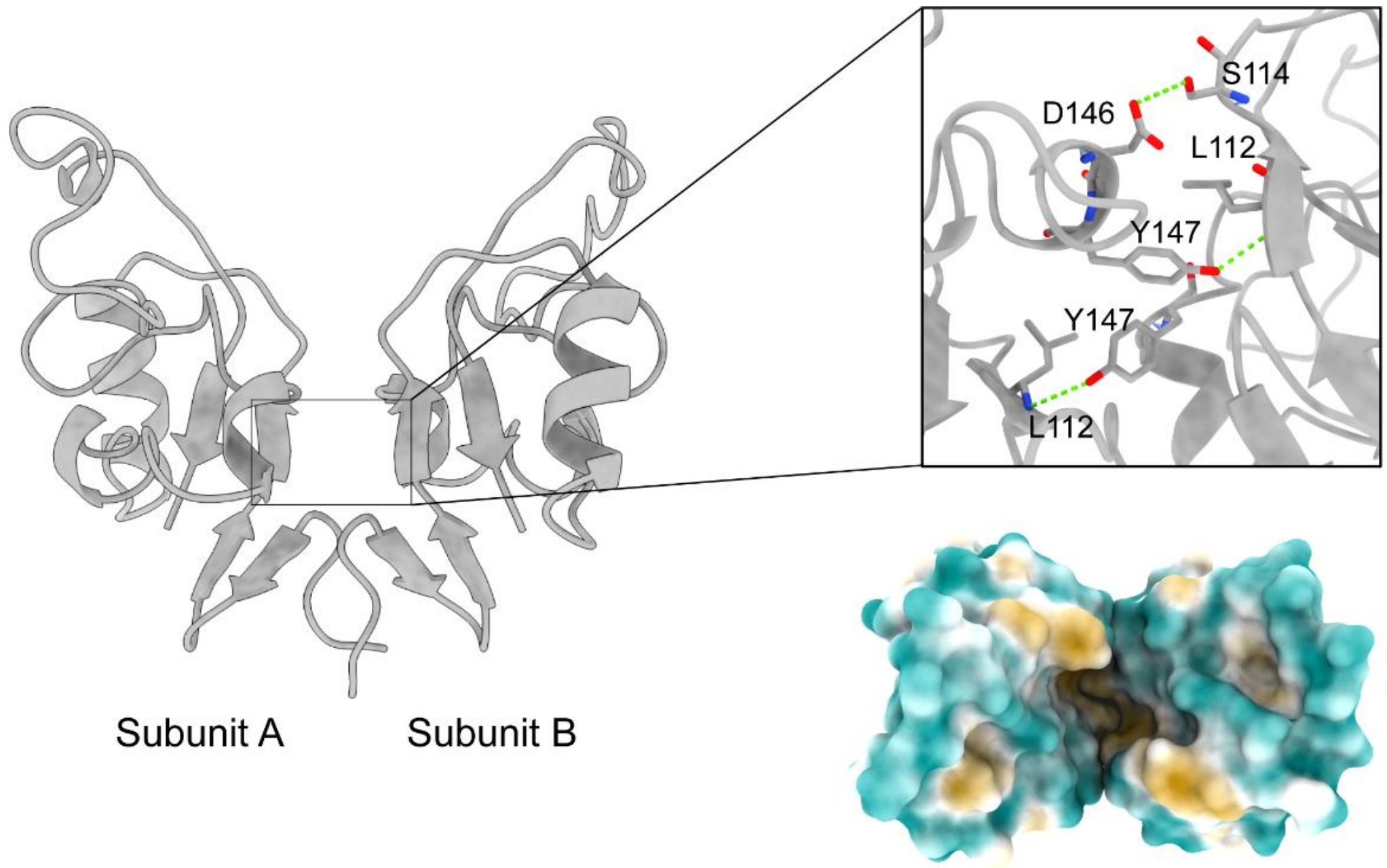

Supplementary Fig. 7 | Overall crystal structure of the A35R Dimer. A35R (coloured dark grey) is displayed in cartoon representation. Bottom right, hydrophobic surfaces of the A35R dimer.

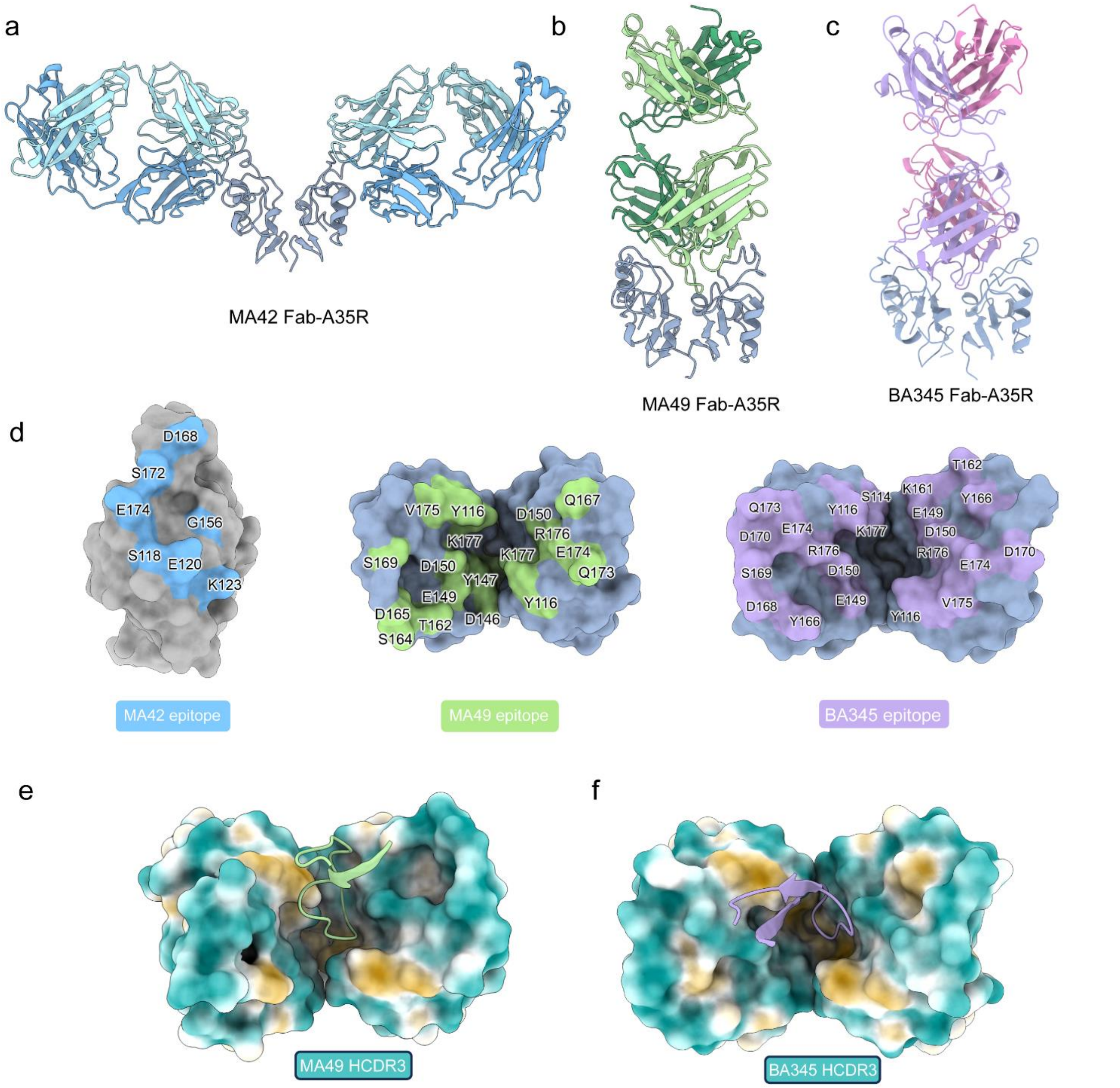

Supplementary Fig. 8 | Crystal structure of the MA42 Fab-, MA49 Fab-, and BA345 Fab-A35R complexes. a, Overall structure of the MA42 Fab/A35R complex. The heavy chain (coloured cyan) and light chain (coloured dodger blue) of the MA42 Fab region and A35R (coloured dim grey) are shown in cartoon representations. **b,** Overall structure of the MA49 Fab/A35R complex. The heavy chain (coloured green) and light chain (coloured blue) of the MA49 Fab region and A35R (coloured dim grey) are displayed in cartoon representations. **c,** Overall structure of the BA345 Fab/A35R complex. The heavy chain (coloured purple) and light chain (coloured pink) of the BA345 Fab region and A35R (coloured dim grey) are displayed in cartoon representations. **d,** Binding epitopes of A35R recognized by MA42, MA49, and BA345. **e,** Interaction interface between the HCDR3 region of MA49 and the

A35R dimer. The HCDR3 regions of MA49 (coloured green) are shown in cartoon representations. **(f)** Interaction interface between the HCDR3 region of BA345 and the A35R dimer. The HCDR3 regions of BA345 (coloured purple) are shown in cartoon representations.

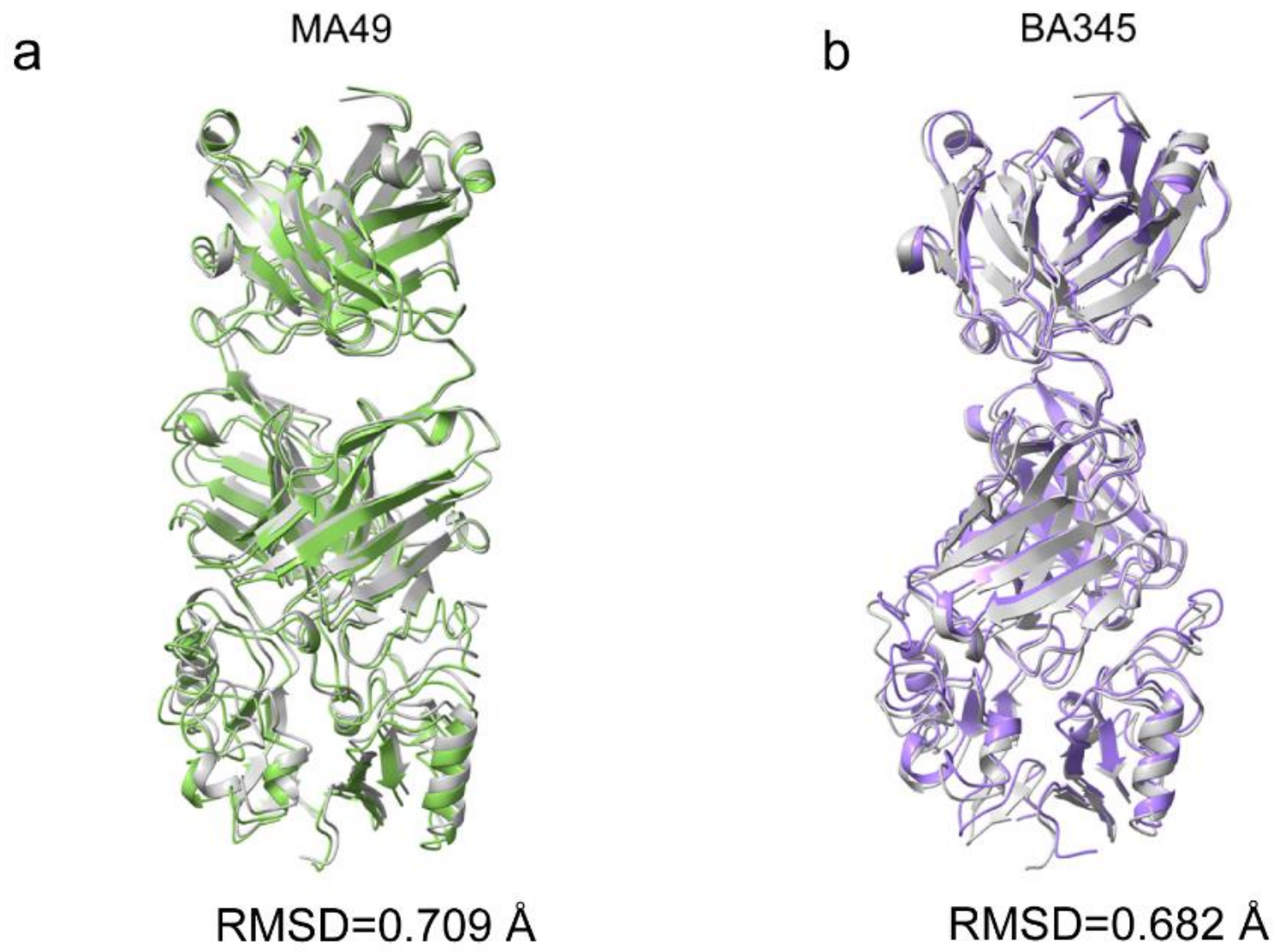

Supplementary Fig. 9 | Comparison of X-ray crystal structures of the MA49 Fab-A35R and BA345 Fab-A35R complexes with the corresponding EnsembleFold-predicted structures. a, Ribbon representation of the MA49 Fab-A35R crystal structure (green) overlaid onto the EnsembleFold-predicted structure (grey). **b,** Ribbon representation of the BA345 Fab-A35R crystal structure (purple) overlaid onto the EnsembleFold-predicted structure (gray).

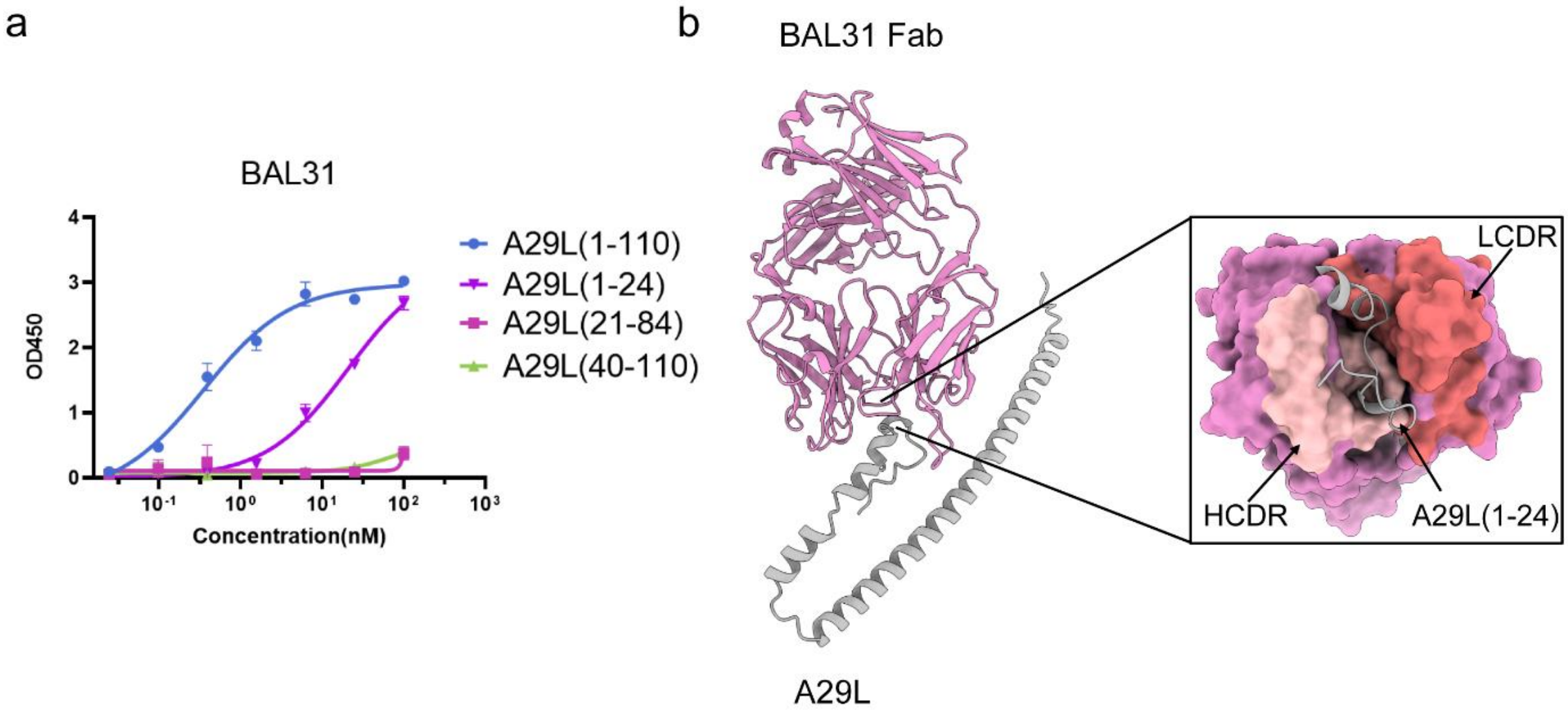

Supplementary Fig. 10 | Epitope mapping for the binding of BAL31 to A29L. a, ELISA binding profiles of BAL31 against the full A29L (1-110) and A29L following N-terminal (1-24 and 21-84) and C-terminal truncations (40-110). **b,** Predicted structure of the BAL31 Fab-A29L (1-110) complex. Left: Ribbon representation with the BAL31 Fab region in pink and A29L in grey. Right: Molecular surface representation highlighting heavy chain CDRs (HCDRs, red) and light chain CDRs (LCDRs, blue).

Supplementary Table 1. Patient sample information

Patient	Sex	Age	Disease severity	Symptoms	Recovery time (days)	Sample collection time (days after discharge)
Donor 1	Male	20	Mild	Rash, fever	16	17
Donor 2	Male	43	Mild	Rash, fever	30	15
Donor 3	Male	25	Mild	Rash	13	12
Donor 4	Male	38	Mild	Rash, fever	18	5
Donor 5	Male	63	Mild	Rash	13	6

Supplementary Table 2. Crystal data collection and refinement statistics.

(MA42 Fab/A35R PDB ID:9UDQ)

Wavelength (Å)	0.96183
Resolution range (Å)	31-2.82 (2.92-2.82)
Space group	$P\ 2_1\ 2_1\ 2$
Unit cell a, b, c (Å) and α, β, γ (°)	147.483, 185.993, 41.753, 90, 90, 90
Total reflections	150,898
Unique reflections	28658 (1380)
Multiplicity	5.3 (5.2)
Completeness (%)	97.62 (93.49)
Mean I/sigma (I)	9.4 (2.25)
Wilson B-factor (Å²)	43.62
R-meas (%)	17.6 (61.5)
R-pim (%)	7.6 (26.5)
CC1/2	0.958 (0.865)
Reflections used in refinement	28,001 (2,599)
Reflections used for R-free	1,998 (186)
R-work (%)	21.26 (29.28)
R-free (%)	27.62(36.08)
Number of nonhydrogen atoms	7888
Macromolecules	7878
Ligands	0
Solvent	10
Protein residues	1036
RMS (bonds) (Å)	0.011
RMS (angles) (°)	1.28
Ramachandran favoured (%)	95.80
Ramachandran allowed (%)	4.20
Ramachandran outliers (%)	0.00
Rotamer outliers (%)	2.82
Clashscore	15.62
Average B-factor (Å²)	43.10
Macromolecules	43.11

Data for the highest-resolution shell are shown in parentheses.

Supplementary Table 3. Crystal data collection and refinement statistics.

(MA49 Fab/A35R PDB ID:9UE0)

Wavelength (Å)	0.97861
Resolution range (Å)	44.95-2.70 (2.80-2.70)
Space group	$P\ 2_1\ 2_1\ 2_1$
Unit cell a, b, c (Å) and α, β, γ (°)	81.766, 82.528, 107.19, 90, 90, 90
Total reflections	266,002
Unique reflections	20,552 (998)
Multiplicity	12.9（12.8）
Completeness (%)	99.58 (98.45)
Mean I/sigma(I)	11.5（2）
Wilson B-factor (Å²)	43.42
R-meas (%)	28.6（91.7）
R-pim (%)	7.8（25.1）
CC1/2	0.844（0.827）
Reflections used in refinement	20,285 (1,928)
Reflections used for R-free	1995 (185)
R-work (%)	22.26 (31.16)
R-free (%)	27.22 (40.05)
Number of nonhydrogen atoms	4,682
Macromolecules	4,664
Ligands	0
Solvent	18
Protein residues	605
RMS (bonds) (Å)	0.005
RMS (angles) (°)	0.97
Ramachandran favoured (%)	95.48
Ramachandran allowed (%)	4.52
Ramachandran outliers (%)	0.00
Rotamer outliers (%)	3.24
Clashscore	5.17
Average B-factor(Å²)	42.99
Macromolecules	43.04

Data for the highest-resolution shell are shown in parentheses.

Supplementary Table 4. Crystal data collection and refinement statistics

(BA345 Fab/A35R PDB ID:9UE8)

Wavelength (Å)	0.96183
Resolution range (Å)	47.97-3.20 (3.31-3.20)
Space group	$P\ 2_1\ 2_1\ 2_1$
Unit cell a, b, c (Å) and α, β, γ (°)	113.063, 126.067, 350.292, 90, 90, 90
Total reflections	921,563
Unique reflections	73,823 (3610)
Multiplicity	12.5（10.9）
Completeness (%)	99.56 (98.17)
Mean I/sigma(I)	5.6（1.7）
Wilson B-factor (Å²)	43.17
R-meas (%)	48.9（129.4）
R-pim (%)	13.7（38.1）
CC1/2	0.926（0.832）
Reflections used in refinement	83,150 (8,120)
Reflections used for R-free	2000 (196)
R-work (%)	21.51 (28.35)
R-free (%)	26.98 (33.39)
Number of nonhydrogen atoms	27,666
Macromolecules	27,666
Ligands	0
Solvent	0
Protein residues	3624
RMS (bonds) (Å)	0.013
RMS (angles) (°)	1.71
Ramachandran favoured (%)	95.05
Ramachandran allowed (%)	4.95
Ramachandran outliers (%)	0.00
Rotamer outliers (%)	2.69
Clashscore	16.55
Average B-factor (Å²)	43.56
Macromolecules	45.56

Data for the highest-resolution shell are shown in parentheses.

Supplementary Table 5. PISA analysis of the interactions between the MA42 Fab, MA49 Fab, and BA345 Fab regions and A35R

NAb	A35R	Interface area, Å^2	ΔiG (kcal/m)	ΔiG (P value)	NHB	NSB	NDS	CSS
MA42	HC	494.3	-2.2	0.619	13	1	0	0.186
	LC	291.1	-1.4	0.529	7	0	0	0.103
MA49	HC	1063.6	-1.1	1.376	18	0	0	0
	LC	370.6	0.9	1.372	7	0	0	0
BA345	HC	1397.9	-6.5	1.186	26	6	0	0.762
	LC	499.4	0.2	1.195	9	0	0	0.08

Table S6. Residues contributing to the interaction between the MA42 Fab region and A35R

MA42 Fab	Distance (Å)	A35R
Hydrogen Bonds		
VH:THR 28[N]	3.34	J:SER 169[O]
VH:THR 28[OG1]	3.18	J:ASP 170[O]
VH:THR 28[OG1]	3.57	J:SER 169[O]
VH:SER 31[OG]	3.90	J:ASP 170[O]
VH:ARG 100[NH1]	3.47	J:GLY 156[O]
VH:ARG 100[NH2]	3.78	J:GLU 120[OE2]
VH:ARG 101[NH2]	2.10	J:GLY 156[O]
VH:GLY 102[N]	3.76	J:SER 172[O]
VH:ASN 104[N]	2.83	J:GLU 174[OE1]
VH:ASN 104[ND2]	2.80	J:GLU 174[OE2]
VH:TYR 105[N]	2.66	J:GLU 174[OE1]
VH:TYR 106[N]	2.98	J:GLU 174[OE1]
VH:TYR 106[OH]	3.58	J:ASP 121[OD2]
VL:ASN 31[ND2]	2.83	J:GLU 120[O]
VL:TYR 32[OH]	2.52	J:SER 118[OG]
VL:TYR 32[OH]	2.46	J:ASP 121[OD1]
VL:THR 53[OG1]	3.44	J:SER 154[O]
VL:TYR 91[OH]	3.03	J:GLU 120[OE2]
VL:TYR 32[OH]	3.86	J:ASP 121[N]
VL:ALA 50[O]	2.65	J:LYS 123[NZ]
Salt bridges		
F:ARG 100[NH2]	3.78	J:GLU 120[OE2]

Table S7. Residues contributing to the interaction between the MA49 Fab region and A35R

MA49 Fab	Distance (Å)	A35R
Hydrogen Bonds		
A:ASN 57[OD1]	3.30	D:VAL 175[N]
A:THR 58[O]	2.72	D:SER 169[OG]
A:TYR 103[O]	3.64	D:TYR 116[OH]
A:TYR 103[O]	3.10	D:LYS 177[NZ]
A:ASN 55[ND2]	3.53	D:VAL 175[O]
A:ASN 57[ND2]	2.72	D:VAL 175[O]
A:TYR 102[OH]	2.71	D:ASP 150[OD2]
A:GLY 106[N]	2.63	D:GLU 149[O]
A:GLY 106[N]	3.33	D:TYR 147[O]
A:SER 107[N]	3.85	D:ASP 146[O]
A:SER 107[OG]	3.75	D:ASP 146[OD1]
A:PHE 101[O]	2.46	C:TYR 116[OH]
A:TYR 32[OH]	3.54	C:ARG 176[NH2]
A:TYR 103[OH]	3.48	C:LYS 177[N]
A:SER 105[O]	3.58	C:LYS 177[NZ]
A:TYR 103[OH]	2.46	C:ASP 150[O]
A:TYR 32[OH]	2.99	C:GLU 174[OE1]
A:THR 28[OG1]	3.40	C:GLN 167[NE2]
B:THR 56[OG1]	2.11	C:GLN 173[NE2]
B:TYR 49[OH]	3.24	C:TYR 116[O]
B:ASN 93[OD1]	3.88	D:ASP 165[N]
B:ASN 93[OD1]	2.85	D:SER 164[OG]
B:ASN 93[OD1]	3.19	D:SER 164[N]
B:ASN 93[OD1]	2.91	D:THR 162[OG1]
B:TRP 94[O]	2.94	D:SER 164[OG]

Table S8. Residues contributing to the interaction between the BA345 Fab region and A35R

BA345 Fab	Distance (Å)	A35R
Hydrogen Bonds		
U:LEU 1[N]	2.78	W:SER 169[OG]
U:LEU 1[N]	2.94	W:ASP 170[O]
U:VAL 2[N]	3.00	W:GLU 174[OE2]
U:VAL 2[N]	3.21	W:GLU 174[OE1]
U:ARG 97[NH2]	3.40	W:GLU 174[OE2]
U:ARG 100[N]	3.23	W:TYR 116[OH]
U:ARG 100[NE]	3.16	W:TYR 116[OH]
U:ARG 100[NH1]	3.20	W:GLU 149[O]
U:ARG 100[NH2]	3.87	W:LYS 177[O]
U:ARG 100[NH2]	2.90	W:ASP 150[O]
U:GLY 26[O]	3.50	W:ASP 168[N]
U:GLY 26[O]	2.74	W:SER 169[OG]
U:SER 32[OG]	3.67	W:ARG 176[NH1]
U:THR 73[O]	2.81	W:TYR 166[OH]
U:ARG 100[O]	2.94	W:TYR 116[OH]
U:SER 56[OG]	3.52	X:TYR 116[O]
U:ASN 58[ND2]	2.73	X:VAL 175[O]
U:ARG 64[NH1]	3.60	X:ASP 170[OD2]
U:ARG 64[NH2]	3.88	X:ASP 170[O]
U:ARG 64[NH2]	3.85	X:GLU 174[OE2]
U:SER 102[OG]	3.88	X:ASP 150[OD2]
U:SER 106[N]	3.20	X:ASP 150[OD2]
U:SER 106[OG]	3.16	X:ASP 150[OD2]
U:LEU 107[N]	3.47	X:ASP 150[OD2]
U:ASN 58[OD1]	2.96	X:VAL 175[N]
U:SER 106[OG]	2.63	X:ARG 176[NH1]
V:SER 30[OG]	3.35	X:THR 162[OG1]
V:SER 30[OG]	2.85	X:TYR 166[OH]
V:TYR 33[OH]	3.06	X:GLU 149[OE2]
V:SER 95[OG]	2.72	X:GLU 174[OE1]
V:TYR 33[OH]	3.66	X:LYS 161[NZ]
V:GLY 93[O]	2.82	X:TYR 166[OH]
V:ARG 54[NH1]	3.51	W:TYR 116[O]
V:ARG 54[NH2]	3.56	W:SER 114[O]
V:THR 57[OG1]	3.64	W:GLN 173[O]
Salt bridges		
U:LEU 1[N]	3.30	W:GLU 174[OE1]

U:ARG 97[NH1]	3.27	W:GLU 174[OE2]
U:ARG 97[NH1]	3.83	W:GLU 174[OE1]
U:ARG 97[NH2]	3.40	W:GLU 174[OE2]
U:ARG 64[NH1]	3.60	X:ASP 170[OD2]
U:ARG 64[NH2]	3.85	X:GLU 174[OE2]

Table S9. BSA (Å^2) of each protomer of A35 (subunits A and B) buried by heavy and light chains of the mAbs

	BA345/A35R	EV35-2/A35R	EV35-6/A35R	EV35-7/A35R	mAb 975/A35R	mAb 981/A35R
HC	1398.3	970	969	594	983.3	910.8
LC	499.7	302	234	246	514.1	421.9
Totle	1898	1272	1203	840	1497.4	1332.7

Table S10. Comparative analysis of antibody germline and HCDR3 sequences

mAb	Germline	HCDR3
BA345	IGHV4-59	CARRMRASLFGSLGWLDPW
EV35-2	IGHV1-2	CAKIYCRGDCYSYFDYW
EV35-6	IGHV4-34	CARVGGYYYGWGKKPHWFDPW
EV35-7	IGHV3-30	CARQDCGGDCFSRFDSW
mAb 975	IGHV4-4	CARSQGGSGLFRAKWFDPW
mAb 981	IGHV4-39	CARLRLGSLLHRYRAVDPW
